# The complexity of alternative splicing and landscape of tissue-specific expression in lotus (*Nelumbo nucifera*) unveiled by Illumina- and single-molecule real-time-based RNA-sequencing

**DOI:** 10.1093/dnares/dsz010

**Published:** 2019-06-07

**Authors:** Yue Zhang, Tonny Maraga Nyong'A, Tao Shi, Pingfang Yang

**Affiliations:** 1 CAS Key Laboratory of Aquatic Botany and Watershed Ecology, Wuhan Botanical Garden, Chinese Academy of Sciences, Wuhan, CN, China; 2 University of Chinese Academy of Sciences, Beijing, China; 3 State Key Laboratory of Biocatalysis and Enzyme Engineering, School of Life Sciences, Hubei University, Wuhan, China

**Keywords:** SMRT sequencing, Illumina RNA-seq, full length, alternative splicing, isoform variants

## Abstract

Alternative splicing (AS) plays a critical role in regulating different physiological and developmental processes in eukaryotes, by dramatically increasing the diversity of the transcriptome and the proteome. However, the saturation and complexity of AS remain unclear in lotus due to its limitation of rare obtainment of full-length multiple-splice isoforms. In this study, we apply a hybrid assembly strategy by combining single-molecule real-time sequencing and Illumina RNA-seq to get a comprehensive insight into the lotus transcriptomic landscape. We identified 211,802 high-quality full-length non-chimeric reads, with 192,690 non-redundant isoforms, and updated the lotus reference gene model. Moreover, our analysis identified a total of 104,288 AS events from 16,543 genes, with alternative 3ʹ splice-site being the predominant model, following by intron retention. By exploring tissue datasets, 370 tissue-specific AS events were identified among 12 tissues. Both the tissue-specific genes and isoforms might play important roles in tissue or organ development, and are suitable for ‘ABCE’ model partly in floral tissues. A large number of AS events and isoform variants identified in our study enhance the understanding of transcriptional diversity in lotus, and provide valuable resource for further functional genomic studies.

## 1. Introduction

Gene expression is generally defined as the decoding of DNA information into proteins, which includes two basic steps: transcription of DNA into RNA and translation of RNA into protein. Although these two steps occur in the same spot in prokaryotes, they are separated at different subcellular locations in eukaryote.^1^ This separation, along with the complicated gene structure, enables more fine and complex regulations on the eukaryotic gene expression. Among all regulations, alternative splicing (AS), defined as the inclusion of different exons into mature mRNA, is an important mechanism that could dramatically increase the diversity of transcriptome and proteome, and hence regulating different physiological and developmental processes in eukaryote.^2^^,^^3^ Furthermore, AS could also regulate gene expression level and spatio-temporal specificity.^4^ Studies have shown that >90% genes in human and >60% multi-exon genes in Arabidopsis undergo AS.^5^^,^^6^ There are five major AS groups, intron retention (IR), exon skipping (ES), alternative donor (AD), alternative acceptor (AA) and alternative position (AP), of which IR is prevalent in plants,^7^ whereas, ES is more common in mammals.^8^^,^^9^

Although the functions of most multiple isoforms generated by AS remain uncovered, several studies have demonstrated the biological importance of AS in different physiological and developmental processes. Studies showed that the *Bcl-x* gene in *Drosophila* could produce two isoforms playing contradicting roles with one activating apoptosis while the other one inhibiting apoptosis.^10^ Studies have also shown the function differentiation of the same genes with different transcripts in plants. In Arabidopsis, AS of *zinc-induced facilitator-like 1* (*ZIFL1*) gene could generate two isoforms: *ZIFL1.1* and *ZIFL1.3*, of which the former influences polar transportation of auxin in roots, whereas the latter regulates the movement of stomatal.^11^ In other examples, different transcripts of *YUCCA4* show different features with respect to subcellular localization and tissue specificity,^12^ and also different *PHYTOCHROME INTERACTING FACTOR6* (*PIF6*) transcripts in the ABA-dependent seed germination play different functions.^13^ Different AS isoforms are involved in stress response and circadian in Arabidopsis.^14–17^ Similar discoveries have also been obtained in cereals, such as the *Rough endosperm3* (*Rgh3*) gene (>19 transcript isoforms) of maize involved in endosperm differentiation and two transcripts of *OsDR11* gene of rice involved in the process of disease resistance.^18^^,^^19^

Due to the biological importance of AS and the reduction of sequencing cost, comprehensive surveys of AS have been conducted widely in plants using next-generation sequencing (NGS) technology, which include Arabidopsis and other types of crops.^20–22^ Although, the NGS has been proven to be powerful, it still faces limitation in accurate identification of each isoform because of its short sequencing reads,^23^ which is much more serious in polyploid species or species with abundant repetitive genome sequences. However, with the advent of PacBio single-molecule real-time (SMRT) sequencing technology, known as the third generation of sequencing technology, full-length sequence of mRNA could be obtained without *in silico* sequence assembly.^24^ Because of its ability to generate much longer reads, it can easily detect the existence of different transcripts in each gene, making it appropriate in gene annotation and AS analysis compared with the new-generation sequencing technology.^25^ This SMART technology has been successfully applied in the analysis of AS in different plant species, and emerged to be efficient in enhancing the accuracy of AS identification.^26–30^

Lotus (*Nelumbo nucifera*), which belongs to the family of *Nelumbonaceae*, is a basal eudicot.^31^ It is a crucial species in plant phylogenetics study because it is a slowly evolved and early branching eudicot and retained many ancestral phenotypic features similar to monocots.^32^^,^^33^ Besides, it is also an important aquatic horticultural plant,^34^ thus increasing the necessity to get a better comprehension of lotus. After the successful sequencing and annotation of the genome of two wild strains of sacred lotus, ‘China Antique’ and ‘Chinese Tai-zi’,^33^^,^^35^ the genome database for ‘China Antique’ has been constructed.^36^ However, the genome is poorly annotated because of the short reads and redundant repeat sequences. Although the construction of high resolutions genetic map could aid in genome assembly, more information at transcription level is required to achieve a fine annotation.

To get a comprehensive survey of AS events in sacred lotus, we applied both PacBio SMRT and Illumina-based sequencing of the transcriptome from 16 different tissues of ‘China Antique’. The isoforms identified by both techniques were compared, and the tissue and developmental stage specific AS transcripts were identified and annotated in different tissues. The findings showed the complexity of AS events, while providing comprehensive transcriptome data for further genome annotation on sacred lotus.

## 2. Materials and methods

### 2.1. Plant materials

*Nelumbo nucifera* ‘China Antique’ was cultivated in Wuhan Botanical Garden, CAS (114^°^30ʹE, 30^°^60ʹN) China. Root, leaf, petiole, and rhizome (elongation zone, internode, and apical meristem) tissues, which were used for sequencing, were collected. Samples of petal, receptacle, immature stamen, and unpollinated carpel were sampled on the blooming day (0 d post-anthesis, DPA), while samples of pollinated carpel were collected from 12 h after pollination. Mature stamen and receptacle at 2 DPA, seed-coat at 6, 12, 18 DPA, and cotyledon at 9, 12, and 15 DPA were collected. In total, there are 19 different samples which were immediately frozen in liquid nitrogen for further RNA extraction.

### 2.2. RNA preparation and Illumina RNA-seq

For each sample, total RNA was extracted using the RNAprep pure Plant Kit (TIANGEN). After quality checking by 1% agarose gels, the RNA concentration and integrity were examined using an Qubit^®^ RNA Assay Kit in a Qubit^®^ 2.0 Flurometer (Life Technologies, CA, USA) and an Agilent 2100 Bioanalyzer (Agilent Technologies, CA, USA), respectively. Eligible RNA of each sample (3 μg) was used for constructing the Illumina sequencing library following the recommendations of NEBNext^®^ Ultra™ RNA Library Prep Kit for Illumina^®^ (NEB, USA). The library was sequenced on the Illumina HiSeq 2000 platform at Novogene company (Nanjing, China) and 150 bp paired-end reads were generated.

### 2.3. Single-molecule sequencing of pooled RNA samples

In order to obtain the complete information of full-length transcriptome, total RNA from 19 different samples were pooled together in an equal quantity to construct libraries for PacBio sequencing. The mixed RNA sample was reverse transcribed using the Clontech SMARTer polymerase chain reaction (PCR) cDNA Synthesis Kit and oligo (dT). Large-scale PCR amplification was carried out to generate barcode full-length cDNA. The BluePippin Size Selection System protocol as described by Pacific Biosciences (PN 100-092-800-03) was used to separate the size of PCR selection for mix sample >4 kb. After damage repairing and end joining, the full-length cDNA is ligated to the SMRT dump bell joint. These cDNA products were purified for Iso-Seq SMRTBell library preparation. A SMRT cells were sequenced on the PacBio Sequel System platform.

### 2.4. PacBio reads mapping

The raw data generated from PacBio Sequel sequencing system was processed using the SMRTlinks 5.0 software with min-Length of 200 and min-Read-Score of 0.75. The Circular Consensus sequences (CCSs) were generated from subreads with pass 1 and accuracy >0.8. According to whether the 5ʹ- and 3ʹ-cDNA primers were present and whether there was a poly-A tail signal preceding the 3ʹ-primer, a CCS or subread sequence was divided into full length, non-full length, and chimeric reads. The method of Iterative Clustering for Error Correction (ICE) was employed for iterative clustering to obtain the consensus reads. Polished consensus reads were acquired from the original consensus corrected with non-full-length read using Arrow. To improve consensus accuracy, we use Illumina RNA-seq data to correct the consensus reads with LoRDEC.^37^ Aligning high-quality full-length consensus reads to the reference using GMAP^38^ with >85% alignment coverage and >90% alignment identity. PRAP,^39^ a one-stop solution for Iso-Seq analysis, was used to identify alternative transcription initiation (ATI), AS, alternative cleavage and polyadenylation (APA) by full-length consensus reads. And TACO^40^ was used to combine transcripts from SMRT and Illumina RNA-seq into a consensus merged transcriptome and TransDecoder v5.3.0 was applied to predict the open reading frame. EVidenceModeler (EVM)^41^ was used to predict confident consensus gene models by using the merged transcriptome.

### 2.5. Identification of fusion transcripts

To identify fusion transcripts, a Python script in the PBTRANSCRIPT-ToFU^42^ package was used. The criteria for a candidate fusion transcript was as follows: transcript must be mapped at least two loci in the reference genome and the interval of each loci should be longer than 10 kb; the sequence from the mapped loci should cover more than 99% of the fusion transcript, with sequence from each locus in the fusion transcript covering at least 10%. To further obtain high-quality candidates, the transcripts whose loci in scaffolds other than the nine megascaffolds were filtrated.

### 2.6. Differential expression analysis based on Illumina data

After quality control, clean reads were obtained by removing reads containing adapters, reads containing ploy-Ns and low-quality reads from raw data. The clean reads were mapped to the lotus reference genome v1.1^33^ with Tophat2 v2.1.0 with the default setting. Cufflinks v2.2.1 was used to quantify the expression level of genes and isoforms using the GTF annotation file generated by SMRT and RNA-seq merged transcriptome. To explore tissue-bias expression patterns of isoforms, Mean FPKM >0.1 and coefficient of variation of FPKM (standard deviation divided by mean) >2 were used as the threshold to filter isoforms for hierarchical and *k*-means clustering analysis in *R*. The value of *k* was set to 12, which equals to the number of tissues used in this study. Isoforms or genes with significantly differential expression were identified under the threshold FC (fold change) of FPKM >2 by using the Cuffdiff command between pairwise samples. The significant can be either ‘yes’ or ‘no’, depending on whether *P* is greater than the FDR after Benjamini–Hochberg correction for multiple-testing. Comparing with other tissues, the genes or isoforms which showed significantly higher expression were identified as tissue-specific genes or isoforms.

### 2.7. AS analysis

SUPPA^43^ was employed to classify the AS events with the parameter *-f ioe*. It produces seven kinds of local event types including Skipping Exon (SE), Alternative 5ʹ Splice Sites (A5), Alternative 3ʹ Splice Sites (A3), Mutually Exclusive Exons (MX), Retained Intron (RI), Alternative First Exons (AF) and Alternative Last Exons (AL). Quantification of the differential expression by AS events was carried out using the rMATS.^44^ The output from rMATS, included the differential expression of five main AS events as SE, A5, A3, MX, and RI, was filtered for Inclusion Level Difference >5% and FDR <0.01.

### 2.8. Functional annotation and gene ontology enrichment analyses

To investigate the functions of all isoforms, KOBAS2.0^45^ was used to map all the isoform sequence to gene ontology (GO),^46^ KEGG,^47^ KOG, Pfam, Swiss-Prot, TrEMBL, and Nr database. A GOseq R package based on Wallenius non-central hyper-geometric distribution^48^ was run to obtain the significantly enriched GO terms. Concurrently, all lotus genes were searched against Arabidopsis TAIR10 with BLAST (*e*-value <1e−6) to obtain best hits for further functional annotation.

### 2.9. Reverse transcription-PCR and quantitative real-time PCR

Total RNA (1 µg) from samples were used for first strand cDNA with reverse transcription system, and the product of reaction systems was diluted to 200 µL before reverse transcription-PCR (RT-PCR). Primers were designed to span the splicing events using Primer Premier software (v.5.0) (Supplementary Table S1). The PCR fragments were tested in 1% agarose gel and sequenced by Sanger method. The qRT-PCR reactions were performed on the Bio-Rad using the SYBR. The reaction procedure was initiated at 95°C for 10 s, followed by 40 cycles of 95°C for 15 s, 60°C for 30 s, and 72°C for 30 s with fluorescence detection. Relative gene expressions were analysed using 2^−△△CT^ method, with the lotus *β-Actin* gene used as an internal standard.

## 3. Result

### 3.1. Sequencing of *N. nucifera* transcriptome through SMRT

**Figure 1:**
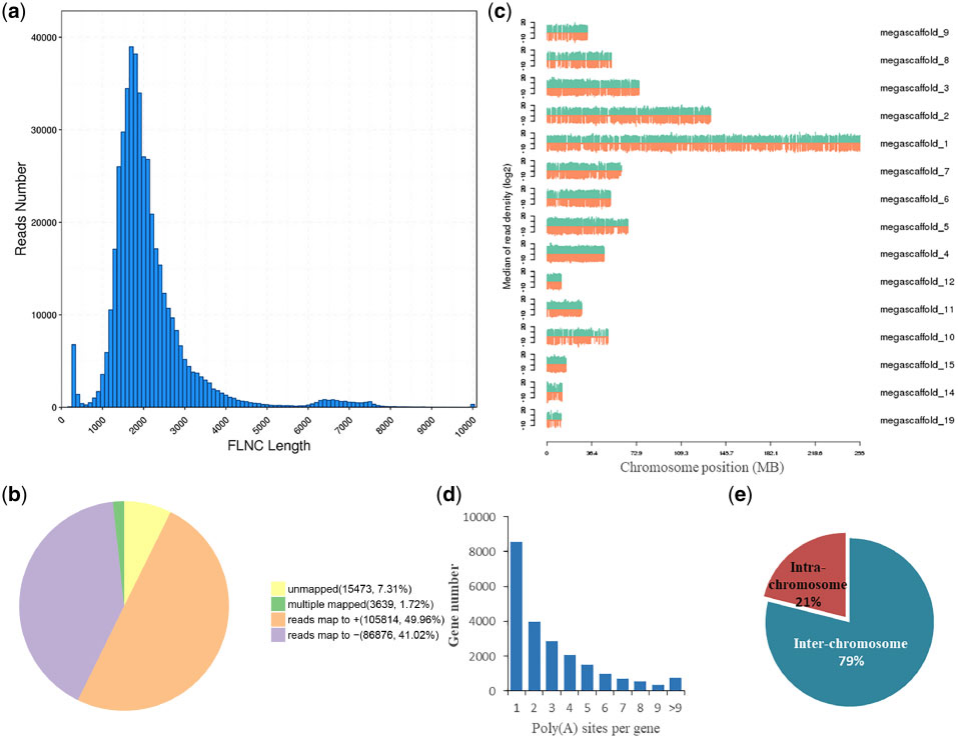
Characterization of lotus transcriptome using Iso-Seq. (a) Density distribution of FLNC reads length obtained by SMART sequencing. (b) The statistics of GMAP mapping by full-length consensus reads. (c) Density distribution of full-length reads in reference genome of lotus. The green is the ‘+’ strand and the orange is the ‘−’ strand. Only the top 10 and other megascaffolds with suitable size and condense mapped reads were shown here. (d) Distribution of the number of poly(A) sites per gene. (e) Ratio of intra- and inter-chromosome fusion transcripts.

In order to obtain a comprehensive full-length transcriptome for *N*. *nucifera*, high-quality RNA was extracted from the above-mentioned 19 different samples including some tissues at different developmental stages. The same amount of RNA per sample was pooled, and size-fractionated library was sequenced on one SMRT cell using PacBio Sequel system. The raw data were then subjected to a series of analyses sequentially (Supplementary Fig. S1). After filtering out low-quality raw reads (length <200 bp and sequence accuracy <0.75) a total of 10,481,648 Reads of Inserts (ROI, 14.55 G) were obtained (Table 1). Further analysis using the Iso-seq protocol generated 670,929 CCSs (Table 1), among which 151,262 and 519,667 were classified as non-full-length and full-length sequences (the reads with 5ʹ primers, 3ʹ primers and poly-A tails), respectively (Supplementary Fig. S1; Table 1). After excluding the chimeric reads, 457,565 full-length non-chimeric (FLNC) reads were clustered using the consensus sequences based on ICE algorithm, accumulating 211,802 polished consensus reads (Table 1). The length of the 211,802 polished consensus reads ranged from 137 bp to 17,645 bp with an average of 2,233 bp (Table 1; Fig. 1a), similar to the 80th percentile value of the reference annotation gene length of 2,184 bp (Supplementary Fig. S2).

**Table 1:** Summary of PacBio Iso-Seq dataset

Category	Dataset
Subreads number	10,481,648
Average subreads length	1,389
Subreads N50	2,034
CCS	670,929
5ʹ-primer	585,493
3ʹ-primer	605,695
Poly-A	588,183
Full length	519,667
FLNC	457,565
Average FLNC read length	2,080
FLNC/CCS	0.68
Polished consensus reads	211,802
Min. length	137
Max. length	17,645
Average consensus reads length	2,233
Polished N50	2,326
Total mapped polished consensus reads	196,329
Multiple mapped reads	3,639
Uniquely mapped reads	192,690
Reads map to ‘+’	105,814
Reads map to ‘−’	86,876

To further improve the accuracy of the consensus reads, Illumina RNA-seq was also applied on the same 19 samples separately. A total of 1.6 billion high-quality short reads were obtained (Supplementary Table S2). Subsequently, we utilized LoRDEC to improve the accuracy of transcripts. The corrected consensus reads were then mapped to the lotus reference genome using GMAP. A total of 192,690 (90.98%) non-redundant isoforms were mapped to unique sequences on the genome, including 105,814 and 86,876 to the ‘+’ and ‘−’ strands, respectively, and 3,639 (1.72%) were mapped to multi-sequences, while the rest were not mapped onto the genome (Table 1; Fig.1b). Moreover, the distribution densities of these full-length consensus reads in reference genome are similar among different megascaffolds, except for megascaffold 4 being the densest one (Fig.1c).

### 3.2. Improving *N. nucifera* genome annotation by SMRT sequencing

**Figure 2:**
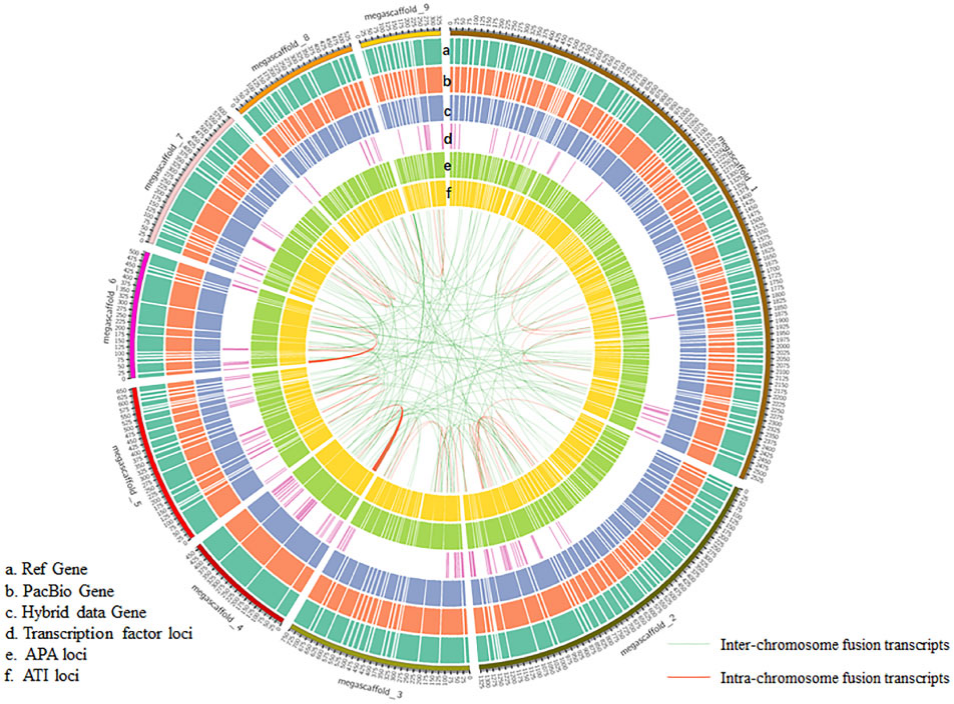
CIRCOS visualization of distribution of different data at genome-wide level. (a) Gene density of reference genome. (b) Density of the predicted genes from PacBio data. (c) Density of predicted genes from hybrid data consisting of PacBio and Illumina data. (d) Distribution of the loci of predicted TF. (e) Distribution of genes with alternative poly(A) site. (f) Distribution of genes with ATI site. Linkage of fusion transcripts was showed in the most inside, with inter- and intra-chromosome, respectively. Based on the mapping results and the distribution of the fusion transcripts, only the biggest nine megascaffolds were shown here.

Considering the advantage of the sequencing length of SMRT technique, much focus has been directed towards the optimization of gene structure and discovery new transcript isoforms, thus leading to the development of various data analysis tools and pipelines, such as TAPIS^27^ and IDP.^49^ Post-transcriptional Regulation Analysis Pipeline for Iso-Seq (PRAPI) was used in the identification of post-transcriptional regulation for its celerity and one-stop operation employed in this study. APA and ATI has been deemed to have capacity for regulating gene expression and increasing transcriptome complexity.^50^ Using the RPAPI, 22,314 genes detected by PacBio have at least one poly(A) site, with 4,850 genes having at least five ploy(A) sites (Supplementary Table S3; Figs 1d and 2). The average number of poly(A) site per gene was 3.07. The ATI events were identified in 11,719 genes detected by PacBio (Supplementary Table S4; Fig. 2). In addition, 271 transcripts mapped at different chromosomes or different loci in the same chromosome were identified as fusion transcripts (Supplementary Table S5; Fig. 2), with the inter-chromosome fusion transcripts being predominant (Fig. 1e).

To obtain comprehensive information of their function, all the isoforms were mapped in different database as described in M&M. The functions of total 32,121 genes identified by Illumina and PacBio data were annotated (Supplementary Table S6). The existing version of lotus reference genome contains 26,685 annotated genes.^33^ In this study, 192,690 unique full-length isoforms were aligned to the reference genome. Further analysis revealed that 19,332 of the reference annotated genes, corresponding to 75,068 transcripts, were supported by one or more full-length reads. A lot of isoforms could not be matched to any of the reference genes, indicating the existence of novel genes. Combining the existing mass of multiple RNA-seq datasets and the new sequencing reads in this study to map against the reference version (Fig. 2), 6,881 novel genes were identified. Approximately one-third (2,456) of the novel genes were supported by both Illumina and PacBio datasets, and the other two-thirds were supported by Illumina (2,298) and PacBio (2,127) data, respectively (Supplementary Fig. S3; Table S7). To verify the existence of these new genes, a total of 20 new genes were randomly selected and identified by RT-PCR (Supplementary Fig. S4). It seems that Illumina-seq and PacBio datasets had nearly similar contribution in discovering novel genes, although the depth of these two was different. To evaluate the homology of these novel genes in Arabidopsis, BLAST analyses were carried out using blastn as described in the Materials and methods section. There are only 396 novel genes with at least one significant match against Arabidopsis cDNA.

In addition, the iTAK software, a programme for classification of plant transcription factor (TF), was used in the identification of TFs by binding to specific *cis*-elements in promoter regions. A total of 7,554 TFs of 50 families were predicted in lotus genome using iTAK tool, and their distribution and gene loci are shown in (Supplementary Fig. S5; Fig. 2). The main TFs identified in our research belong to the MYB (655), AP2 (562), bHLH (531), and C3H (494) families.

### 3.3. Analysis of AS

**Figure 3:**
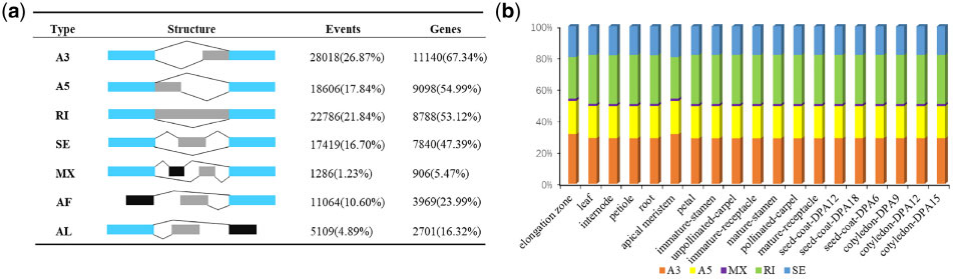
Characterization of AS events and percentage of AS events in tissues. (a) Characterization of AS events. Seven different types of AS events were showed. The number of AS events and corresponding percentage of all AS events were calculated. The number of gene associated with AS event and proportions of these genes occupying all genes undergoing AS. (b) Distribution of five main types of AS events in different tissues.

Based on the method described in M&M, a total of 104,288 AS events were identified from 16,543 reference gene models, accounting for 63.47% of the total annotated protein-coding genes (Supplementary Table S8). To obtain detailed information, the frequency of seven primary AS types, including skipping exon (SE), mutually exclusive exon (MX), alternative 5ʹ splice-site (A5), alternative 3ʹ splice-site (A3), retained intron (RI), alternative first exon (AF), alternative last exon (AL), and the number of corresponding annotated genes were calculated (Fig. 3a). The most abundant AS events in lotus is A3 followed by RI and A5, which is different from other plants having RI as the major AS event. Among the genes regulated by AS, about two-thirds of them (67.33%) were associated with A3 events, and only 5.4% of them were associated with MX events. We found 4,944 (44.38%) of genes regulated by A3 events having one A3 gene loci, the highest number of A3 event identified in a single gene was 38 (Supplementary Table S8). We analysed the AS based on PacBio and Illumina datasets, respectively (Supplementary Fig. S6), in order to gain further insights on the effect of different sequencing techniques on the AS events. Although the number of AS events identified by RNA-seq is more compared with the AS identified by PacBio-Seq, the ratios among different AS events are very similar, with A3 being the major AS event (Supplementary Fig. S6a).

To ascertain the relative importance of the five main models of AS (RI, SE, A3, A5, and MX) in different developmental stages, the RNA-seq datasets of different samples was used to identify AS events, respectively (Fig. 3b). Splicing mode was generally uniform across the 19 samples with little variation on the ratio of different AS events among different tissues. Excluding the two fast growing tissues (elongation zone and apical meristem), RI was the common in all samples, accounting for approximately 31% of alternative transcripts. The number of A3 was the second most predominant. Moreover, the degree of the gene model regulated by AS events in every sample is different, indicating the potential existence of specifically expressive AS events (Fig. 3b, Supplementary Table S8). To filtrate the tissue-specifically expressed AS events (SEASs), rMATS were used in our study.^44^ Among the 12 tissues, a total of 370 SEASs were predicted according to the expression of the splice junctions (Supplementary Fig. S6b; Supplementary Table S9). In particular, the largest number of SEASs found in stamen and carpel tissues, followed by receptacle and seed cotyledon, while no SEASs is identified in the elongation zone. Out of the 370 SEASs, SE was the major one among the five main types of AS events. These results showed that AS might be more preferentially occurring in fast-changing and rapid developing tissues.

**Figure 4:**
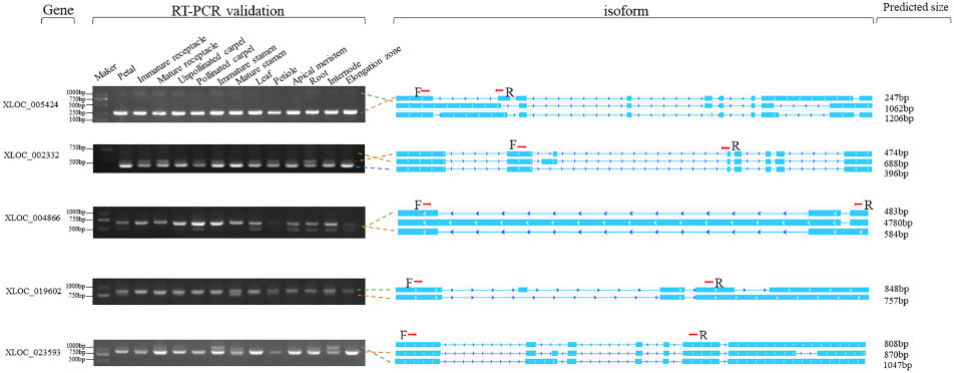
RT-PCR validation of AS events for five genes. Gel bands in each image show DNA makers and PCR results in 13 different samples. Predicted structure of each isoform is shown in right panel. Boxes and line with arrows show exons and introns, respectively. The predicted size of each full length is shown at the end of each line. The loci of PCR primers (F, forward and R, reverse) are shown on the first isoform of each gene.

To avoid the possible interference by false assembly or informatics artefacts, AS events were generated randomly through the selection of 10 genes to perform independent validation by RT-PCR (Fig. 4; Supplementary Fig. S7). We designed primers for the neighbouring regions of these AS junctions and judged the existence of AS events on the basis of the multiple bands. The gel banding pattern and the expected size of the fragments were consistent with the amplified splice isoforms. Some splicing fragments have precedence for high expression in specific tissue. For example, an AS junction from gene *XLOC_025058*, predicted as SEASs, had tissue-preferential high expression in the petal (Supplementary Fig. S7). An isoform from gene *XLOC_019602*, produced by an SE event, exhibited high expression level in the petal, stamen, and petiole but low or none in other tissues (Fig. 4).

### 3.4. Tissue-specific genes and isoforms

**Figure 5:**
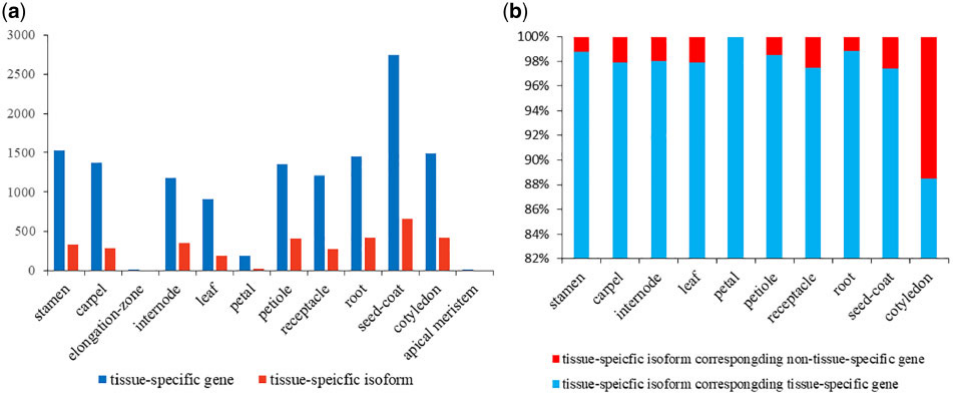
Characterization of tissue-specific genes and isoforms. (a) Distribution of the number of tissue-specific gene and tissue-specific isoform. (b) Percentage of tissue-specific isoforms corresponding non-tissue-specific gene and tissue-specific gene.

One of the primary analyses with Illumina sequencing data involves the identification of differentially expressed genes. Pairwise comparisons among all different tissue samples were performed according to the criteria of fold change >2 and FDR <0.05, to identify the differentially expressed genes and tissue-specific genes. A total of 13,425 genes were identified as tissue-specific genes (Supplementary Table S10), and five randomly selected tissue-specific genes were identified by qRT-PCR (Supplementary Fig. S8). The number of seed-coat-specific genes (2,477) is obviously more than any other tissues (Fig. 5), indicating the unique feature of seed-coat. Interestingly, there are very few elongation zone and apical meristem specific genes (Fig. 5), revealing they are not fully differentiated tissues.

In order to explore the functions of these tissue-specific genes, GO functional enrichment analysis was conducted for these 12 tissues, separately (Supplementary Fig. S9). Tissue-specific genes were classified into three categories (biological processes, molecular functions, and cellular components). In the seed-coat, the most enriched GO terms were ‘regulation of meristem growth’, ‘cysteine biosynthetic process’, ‘response to karrikin’, ‘plant-type cell wall’, and ‘integral component of membrane’. The elongation zone of the rhizome had the least tissue-specific genes, and the functions of these genes were enriched in ‘iron ion binding’ and ‘oxidoreductase activity’. The function of these tissue-specific genes was closely related to the physiological status of the corresponding tissues. For example, in leaf, the most enriched GO terms in cellular component category was ‘chloroplast stroma’, ‘chloroplast thylakoid membrane’, and ‘chloroplast envelope’, otherwise in biological processes category was ‘chlorophyll biosynthetic process’ (Supplementary Fig. S9).

In lotus, previous transcriptomic studies mainly focused on the identification of differential expression genes in specific tissues at different developmental stages.^51^ However, it is clear that most post-transcriptional regulation can change the product of gene expression. So, studies focusing on the level of different isoforms are also important. Based on the criterion described in M&M, the distribution of tissue-specific isoforms generated by AS was analysed in this study to uncover the potential contribution of AS in tissue specialization. Further analyses of 12 tissues revealed that the seed-coat had the most abundant tissue-specific isoforms (662 isoforms), but the elongation zone and apical meristem had none (Fig. 5a). Interestingly, the distribution of tissue-specific isoforms among different tissues had similar patterns with the tissue-specific genes (Fig. 5a). On average, 97.3% of the number of tissue-specific isoforms had their corresponding genes being tissue-specific (Fig. 5b). However, in the cotyledon, the corresponding proportion was 88.5%, lower than other tissues, indicating the possible role of AS in the transcriptional diversity of cotyledon.

GO analyses showed that tissue-specific isoforms are enriched in particular biological processes varying across tissues (Supplementary Fig. S9; Table S11). In the carpel, tissue-specific isoforms were highly enriched in growth and development process, such as ‘response to growth hormone’ and ‘response to karrikin’, consistent with the role of carpel in the seed growth. More importantly, the leaf-specific transcript isoforms were enriched in chlorophyll biosynthetic process and photosynthesis. In contrast, stamen-specific transcript isoforms corresponding genes were enriched in the regulation of double fertilization forming a zygote and an endosperm.

To further understand the isoforms tissue-bias expression patterns, a total of 13,798 isoforms were selected for hierarchical and *k*-means clustering analysis having a mean FPKM >0.1 and standard deviation/mean FPKM >2 (Supplementary Fig. S10). Based on the *k*-means clustering analysis, filtered isoforms were assigned into 12 clusters (Supplementary Fig. S11; Table S12). The isoform levels being clustered to K1 and K8 were enriched in the cotyledon as compared with other tissues. The isoforms being clustered to K4 was enriched in the leaf and the petiole compared with other tissues, indicating strong association of isoforms in K4 to leaf and petiole. Notably, isoforms belonging to all other clusters revealed tissue-biased expression in one or two tissues other than cluster K3.

### 3.5. Differentially expressed genes and isoforms in floral organ specificity

To identify the genes and isoforms associated with the floral development, pairwise comparison among petal, immature stamen, mature stamen, unpollinated carpel, pollinated carpel, immature receptacle, and mature receptacle was conducted. Differential expressed genes and isoforms were identified and classed into three categories: isoform differentially expressed but gene not differentially expressed (type A), both gene and isoform differentially expressed (type B), only gene differentially expressed (type C) (Supplementary Table S13). In total, there were 19,021 genes and 18,399 isoforms identified as differentially expressed (Supplementary Fig. S12). Although there were many differentially expressed isoforms, the number of type A were quite few. This indicates that regulations at isoform level and gene level are mostly correlated, except a few cases (Type A).

**Figure 6:**
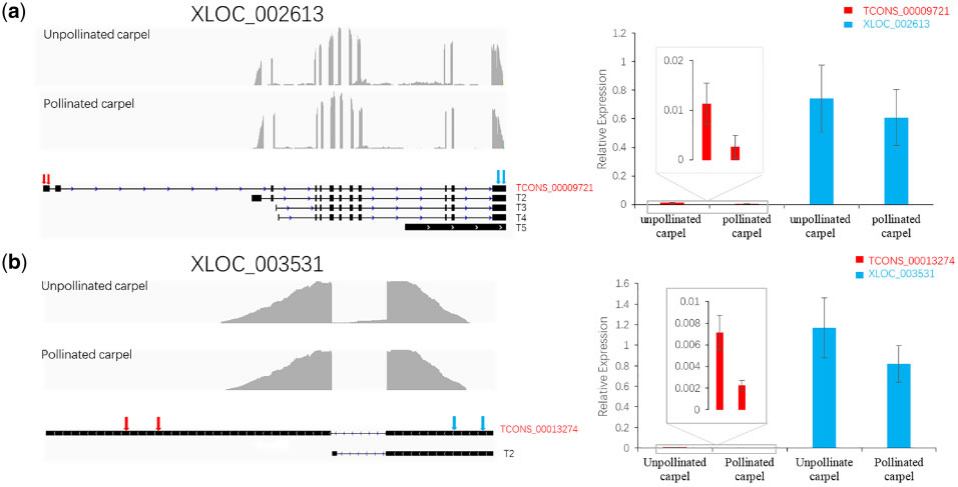
qRT-PCR validation of differential expressed isoforms corresponding gene not differential expressed. (a) Isoform structure of gene XLOC_002613 was shown in left panel; the qRT-PCR results of isoform TCONS_00009721 and gene XLOC_002613 were in right panel. (b) Isoform structure of gene XLOC_003531 was shown in left panel; the qRT-PCR result of isoform TCONS_00013274 and gene XLOC_003531 were in the right panel. Grey peaks indicated read coverage. Black box and line show exons and introns. The region of the two arrows at left side is the specific region of validated isoforms. The region of the two arrows at right side is the comment region of all isoforms, the expression of this region was regarded as expression of this gene. The inserted panels are the enlarged view of the rectangle regions.

To further understand what roles these differential expressed genes and isoforms play in floral organs, GO enrichment analysis was conducted. Between immature and mature stamens, the differential expressed genes were enriched in during floral development, such as ‘inflorescence development’, ‘positive regulation of long-day photoperiodism, flowering’, and ‘response to brassinosteroid’. Between unpollinated receptacle and pollinated receptacle, the function of type A genes was enriched during ‘suspensor development’, ‘actin filament organization’, and ‘response to abscisic acid’, indicating differential expressed transcripts, corresponding gene excluding the differential expressed, had important roles in floral development. To further validate isoforms generated by AS, the RT-PCR experiment was performed to test two isoforms, TCONS_00013274 generated form XLOC_003531 gene and TCONS_00009721 generated from XLOC_002613 gene (Fig. 6).

The ‘ABCE’ model controlling organ specificity has been identified in many plants, and the floral organ genes involved in ‘ABCE’ model have been identified in Arabidopsis.^52–56^ Therefore, we further checked the expression of key ‘ABCE’ genes in lotus base floral organs. Based on the phylogenetic tree analysis of MADS-box gene family from the Arabidopsis and lotus, the lotus ‘ABCE’ model genes were identified (Supplementary Fig. S13). According to the heatmap of MADS-box gene family in lotus, E-class genes were all highly expressed in four floral organ samples, while C-class genes were highly expressed in the carpel (Supplementary Fig. S14). In addition, B-class genes and C-class genes were highly expressed in the stamen, whereas both A- and B-class genes were highly expressed in the petal, consistent with the Arabidopsis model. Additionally, C-class genes were highly expressed in receptacle. These observations provided important insights into the ‘ABCE’ mechanism of lotus floral organ differentiation.

## 4. Discussion

NGS has greatly boosted the understanding of gene structure,^57^ transcriptional network^58^, and small RNAs.^59^ However, the limitation of sequencing length makes the assembly of full-length transcripts challenging, full-length transcript is the approved standard for gene annotation.^60^ In this work, we applied SMRT sequencing in the identification of full-length splice isoforms while investigating the transcriptomic variants in lotus comprehensively, thus overcoming the disadvantage of inaccuracy and difficulty in NGS assembly. The expression of full-length transcripts and loci information would greatly improve the annotation information of lotus genome and provide deep insights into lotus transcriptional landscape.

In order to maximize the transcript diversity and investigate comprehensive splicing isoforms, we broadly harvested 19 samples from 12 tissues/organs. Compared with previous studies in lotus, the largest number of samples was used in our study potentially for covering the most comprehensive transcripts up-to-date. PacBio data from the combined samples might not contain sequencing depth high enough, but the sheer volume Illumina datasets partly made up for exploring low-abundance isoforms when comparing with previous SMRT transcriptomes^27^^,^^30^^,^^61^ studies. Sparse analysis could prove the achieved near-saturation at gene level of the final read depth.^29^ In this study, less than 10% of FLNCs was excavated in the >6,000 bp, indicating the ability of few lotus genes in translating too long FLNCs. Furthermore, the mechanism of fusion transcripts seldom occurred.

Previous transcriptomic study on lotus revealed some novel genes compared with the existing annotation.^62^ However, the annotation information is poor in the existing reference genome, full-length transcriptome resource replenished reference annotation is lacking. The annotation could provide aid in the discovery of novel or previously unrecognized protein-coding genes and isoforms. Through a hybrid sequencing approach combining SMRT and Illumina data, this analysis indicates that full-length transcriptome has a great potential in improving the current lotus annotation. The 6,881 novel genes here account for more than a quarter of the reference genes number, and the number of the genes supported by SMRT data and Illumina data is almost similar. The analysis shows that the initial advantage of discovering novel genes based on Iso-Seq is reduced along with the size of the Illumina sequences getting big enough. Therefore, this hybrid strategy has made considerable trade-off between the cost and efficiency and has been applied extensively in other plants, such as sweet potato,^63^ barley^64^ and *Salvia miltiorrhiza*.^26^

AS is a crucial regulatory mechanism at the post-transcriptional level, since it contributes in transcriptome and proteome diversity.^5^ In lotus, AS events have been identified in four Asian lotus cultivars and the finding revealed alternative 5ʹ first exon as the predominant type of AS events, accounting for 41.2%.^65^ However, it is different with other plant transcriptome study in AS. This study has a comprehensive and systematic analysis of AS in lotus, based on high-quality full-length isoforms and short reads of RNA-seq datasets. Through SMRT and Illumina sequencing, we identified a mass of putative AS events covering 49.3% genes. The ratio is less than 61% reported in Arabidopsis^5^ and 60.5% in cotton.^30^ Alternative 3ʹ splice-site events represent the largest proportion of AS and the number of genes with A3 events occupied 67.33% of all the genes undergoing AS in lotus. This kind of distribution of AS event was inconsistent with other plant studies,^20^^,^^22^^,^^27^ where RI is the major AS event. This research also contradicts with the findings showing the predominance of RI as a mode of AS. In contrast, the RI event was the second largest category based on lotus annotation. Further AS analysis in different tissues indicated the occurrence of A3 events in the elongation zone and apical meristem of rhizome could increase the number of A3 events in the overall AS events. However, this research cannot exclude the possibility of deeper sequencing in identifying other isoforms that update the frequency of different types of AS events. Based on the SEASs analysis,^66^ 94.05% SEASs predominantly occurred in the rapid growing and differentiating tissue, such as seed, carpel, and stamen. This study speculates that the genes regulatory network is complex in these tissues.

This study detected the functional and expressed specificity of both genes and isoforms involved in AS by predicting the annotation and expression quantity. Because the seed-coat features the largest number of tissue-specific genes and the function of these genes are enriched in development and cell wall, there is a possibility that these genes could greatly contribute to the firm structure of the lotus seed-coat which keeps the seed longevity up to a millennium. Our analysis showed that approximately one-quarter tissue-specific isoforms were associated with AS events in per tissue, highlighting AS’ important role in regulating tissue development. In addition, a small part of tissue-specific isoform generated by non-tissue-specific genes as evidenced in this study, excluding the cotyledon, which reveals the strong connection between the expression of tissue-specific isoform and tissue-specific genes. Our result showed that both the function of tissue-specific genes and isoforms were enriched in specific biological processes which vary in different tissues similar to the results reported in other plants.^29^^,^^67^^,^^68^ Additionally, the filtered isoforms showed a tissue-biased expression patterns based on hierarchical and *k*-means clustering analysis other than the tissue-specific isoforms. Therefore, there is a high possibility that the development of tissue or organ was regulated by both the expression level of genes and AS.

Through differential expression analyses conducted in this study, we filtered some differential expressed isoforms, whose corresponding genes were not differentially expressed different from the previous studies. Concurrently, these isoforms were involved in the development of the corresponding tissue samples involved in the key biological process, providing further evidence of the isoforms regulatory functions. In addition, through homologous analyses with Arabidopsis, ‘ABCE’ model genes identified in MADS-box gene family in lotus, proved the compatibility of the ‘ABCE’ model for some floral organs in lotus. Moreover, the intumescent receptacle peculiar to lotus was hypothetically regulated by SHP genes and E-class genes. However, more sampling from different time points in floral organs is needed in investigating the expression of the ‘ABCE’ genes in lotus.

In summary, this study improves the understanding of the transcriptional diversity in lotus by updating the existing gene models and annotations. As transcriptional diversity and complexity is unveiled by both expressional and splicing pattern, a global view of gene regulation in lotus is obtained, thus benefiting gene functional studies and molecular breeding of this economically important aquatic plant.

## 5. Data availability

The Illumina data are accessible through NCBI Sequence Read Archive under accession numbers PRJNA503979 and PRJNA492157. The Iso-Seq sequencing data are accessible through NCBI Sequence Read Archive under accession number PRJNA503979.

## Funding

This study was supported by National Natural Science Foundation of China [grant number 31700197) and Key Research Program of Frontier Sciences, CAS, [grant number QYZDB-SSW-SMC017].

## Conflict of interest

None declared.

## Supplementary Material

dsz010_Supplementary_Data
