# Supplementary material for: The complexity of alternative splicing and landscape of tissue-specific expression in lotus (Nelumbo nucifera) unveiled by Illumina- and single-molecule real-time-based RNA-sequencing
Source: DNA Res. 2019 Jun 7;26(4):301–11. doi: 10.1093/dnares/dsz010 (PMC6704400; doi:10.1093/dnares/dsz010)

Supplementary Figures

**Fig. S1** Flowchart for SMART-sequencing and Illumina RNA-seq data analysis.

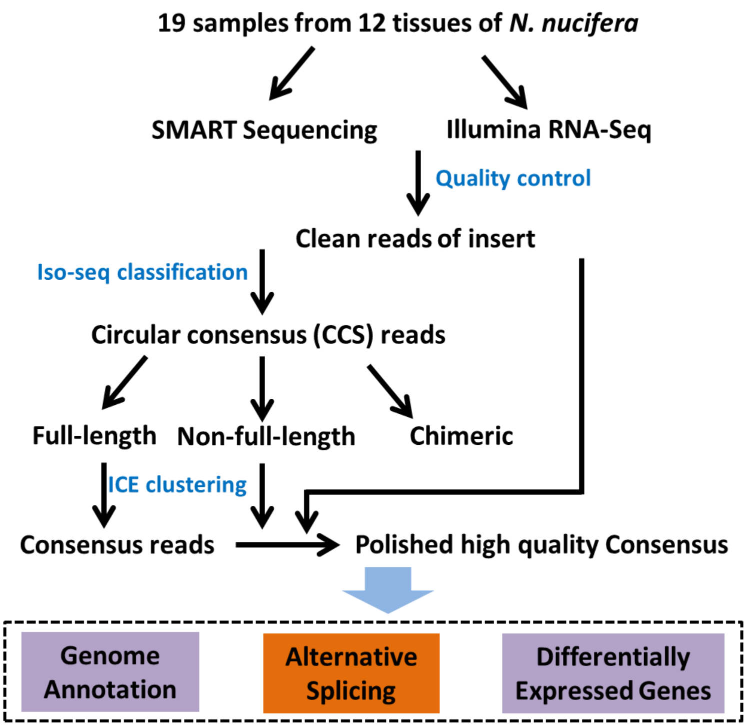


**Fig.S2** Density distribution of reference gene length

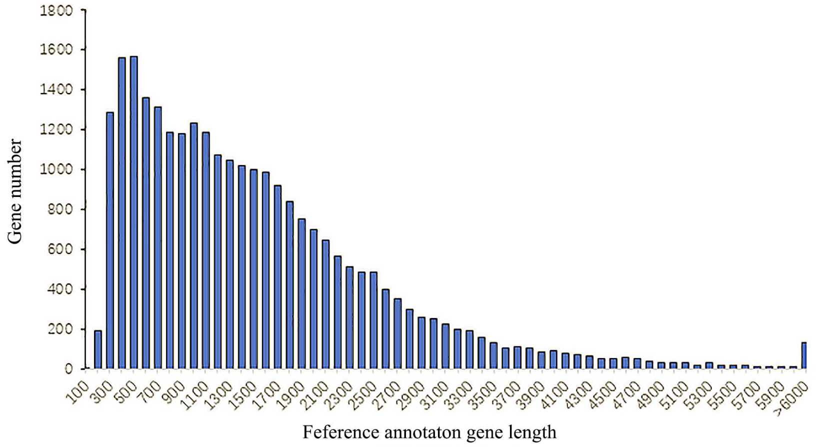


**Fig. S3** Venn diagram showing the common and unique novel genes detected by Illumina and PacBio sequencing.

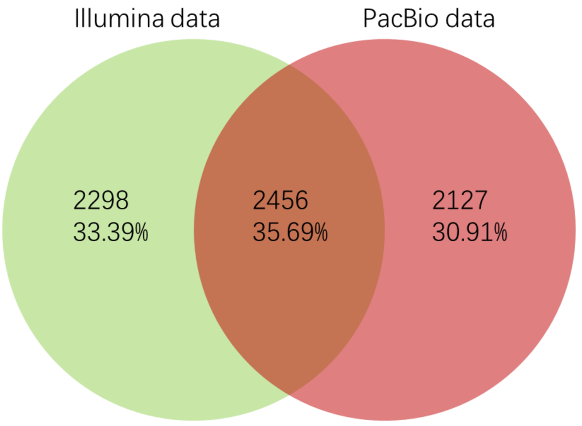

**Fig.S4** RT-PCR validation of 20 new annotation genes

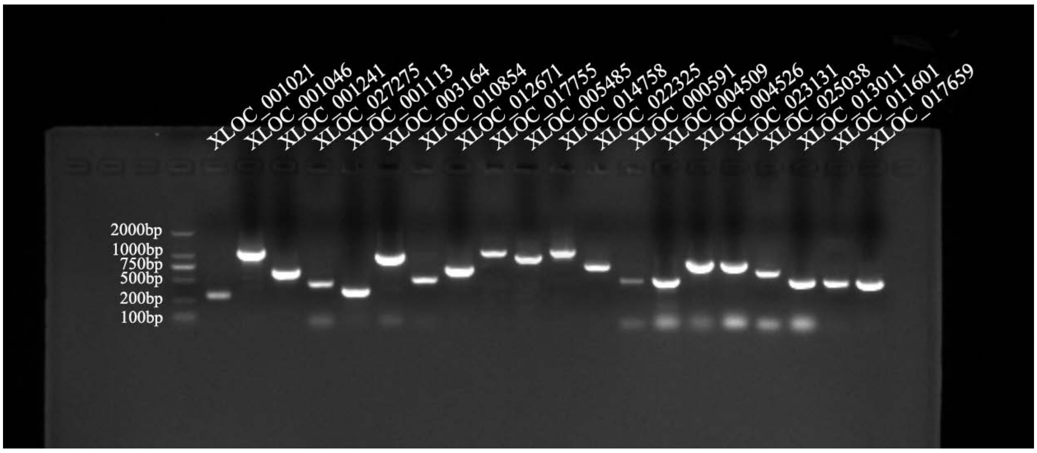


**Fig.S5** The ratio of transcript factors from 50 families

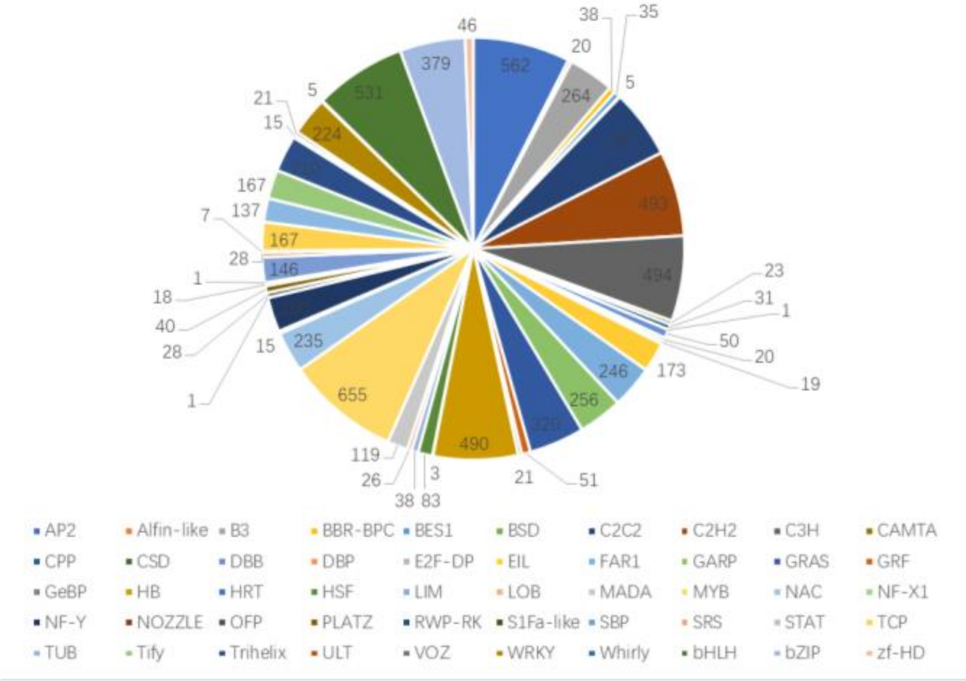

**Fig. S6** Histograms showing the types of AS and their specific distribution among different tissues. (a) The number of five main types of AS events supported by Illumina data and PacBio data, respectively. (b) Distribution of five main types of tissue-specific AS events in twelve lotus tissues.

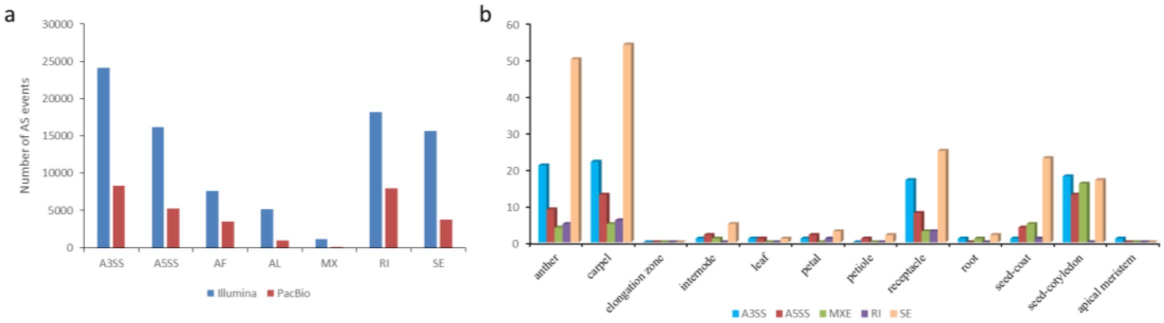


**Fig. S7** RT-PCR validation of AS events for five genes. Gel bands in each image show DNA makers and PCR results in thirteen different developmental stage tissues. Predicted structure of each isoform is shown in right panel. Blue boxes and line with arrows show exons and introns, respectively. The predicted size of each full-length is shown at the end of each line. The loci of PCR primers are shown on the first isoform of each gene.

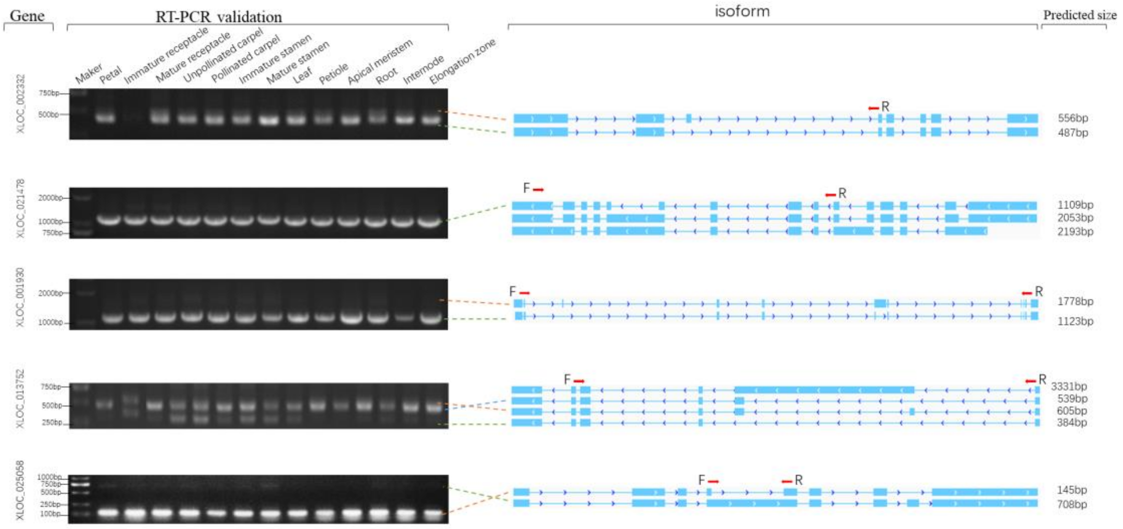

**Fig.S8** qRT-PCR validation of five tissue-specific expressed gene. (a),(b) qRT-PCR result of leaf-specific expressed genes. (c),(d) qRT-PCR result of root-specifc expressed genes. (e) qRT-PCR result of petal-sepcific expressed genes.

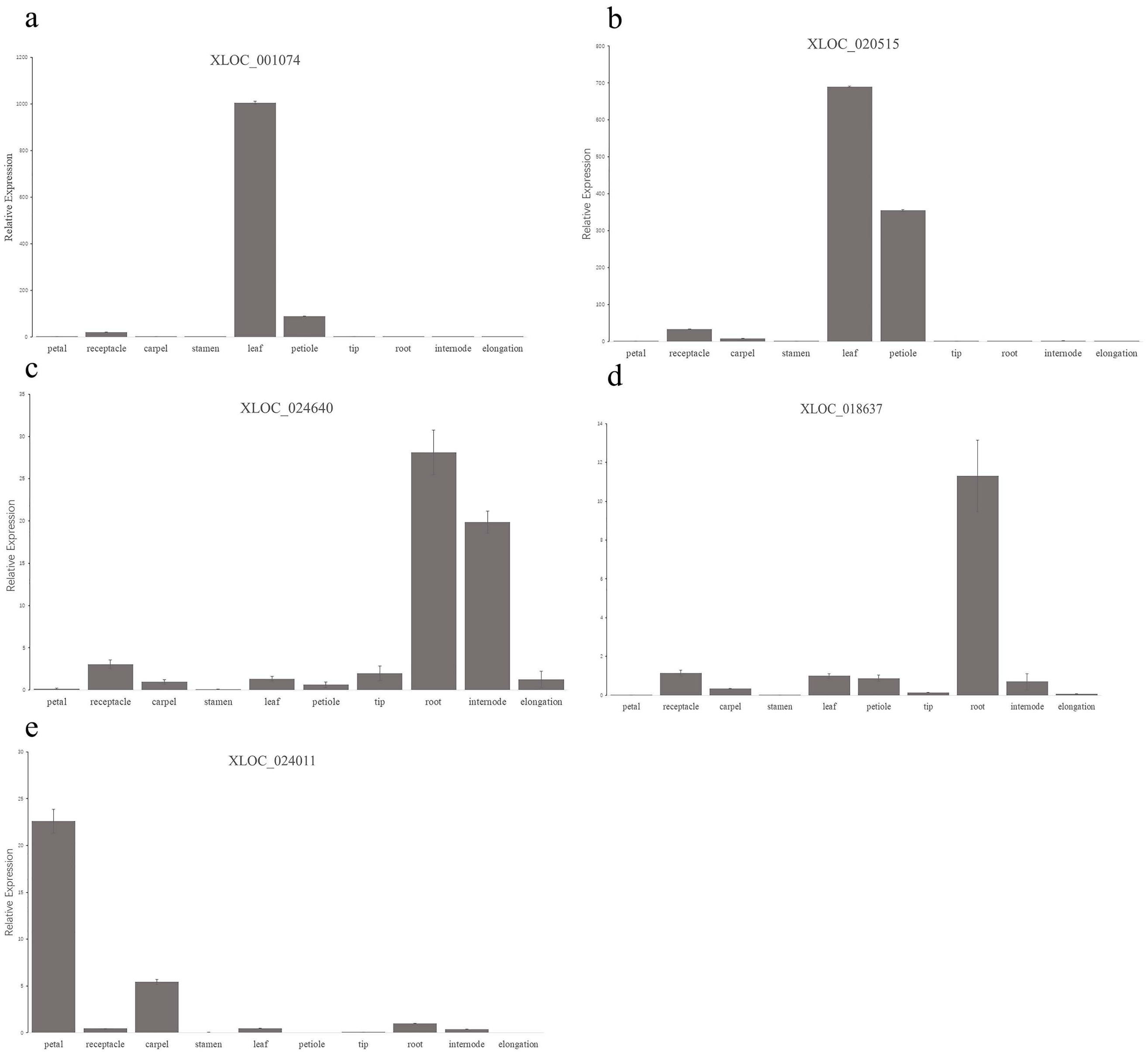

**Supplementary Figure S9** The GO and its enrichment analysis in 12 tissues. a) stamen; b) carpel; c) receptacle; d) cotyledon; e) elongation zone; f) internode; g) apical meristem; h) root; i) leaf; j) petal; k) petiole; l) seed coat. For each image, the up panel showing the distribution of GO functions, and the down panel showing the most enriched functional categories in the corresponding tissue.

Stamen

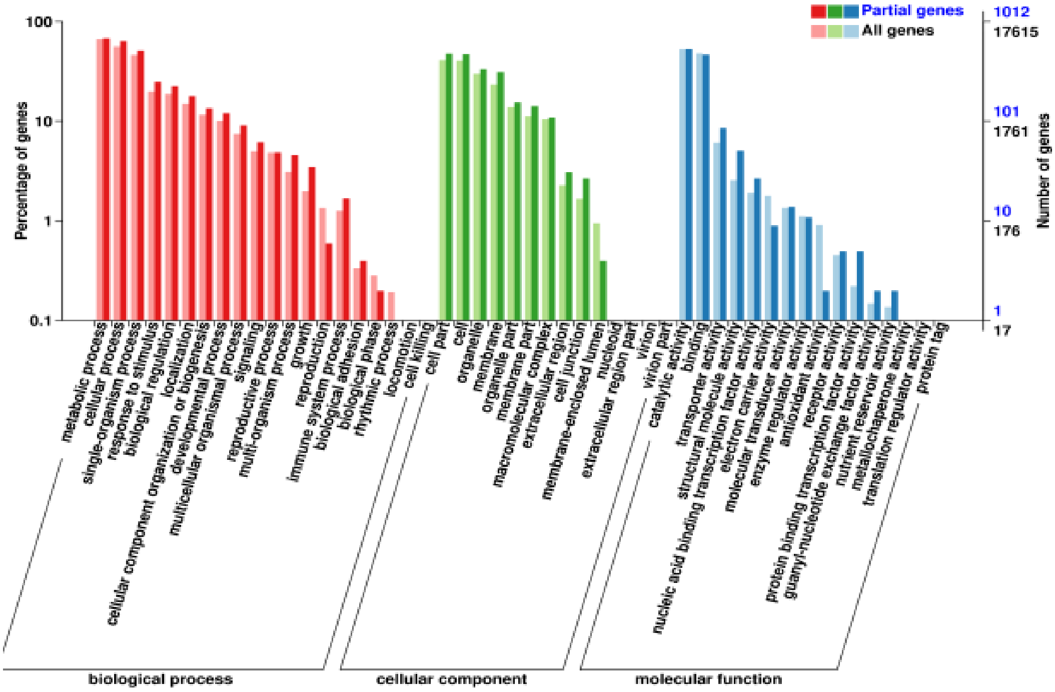


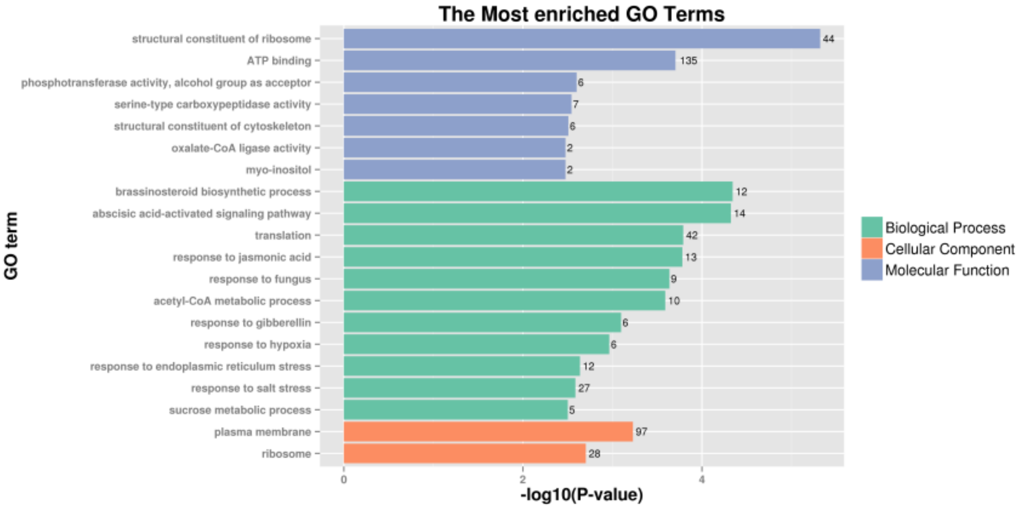

## Carpel

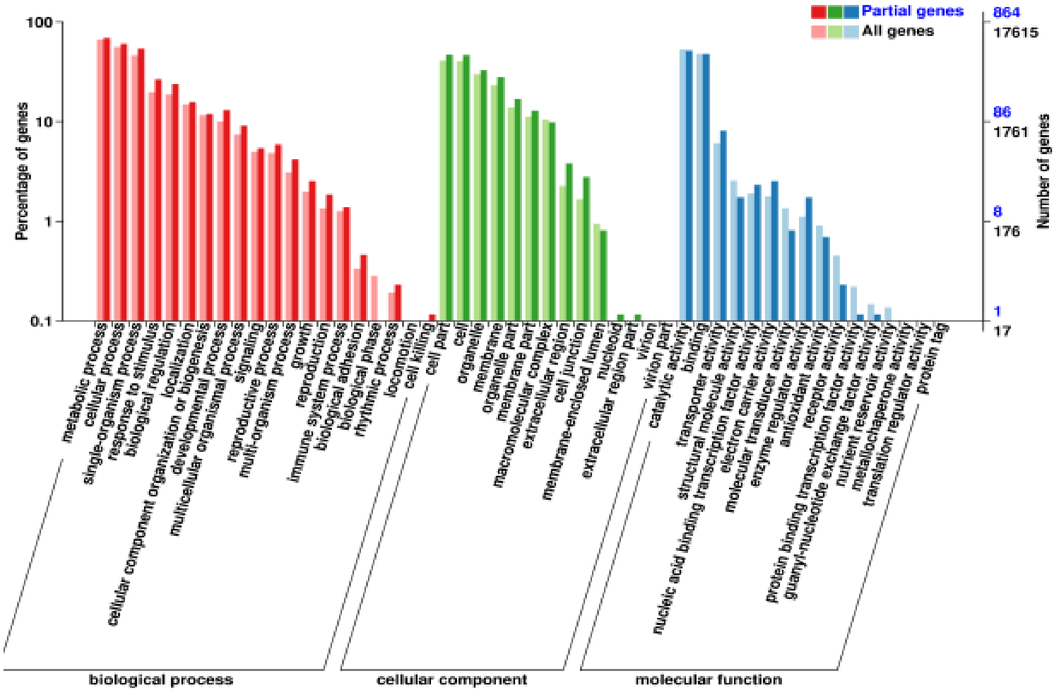

Partial genes
All genes
Percentage of genes
Number of genes
100
10
1
0.1
864
17615
86
1761
8
176
1
17
metabolic process
cellular process
single-organism process
response to stimulus
biological regulation
localization
cellular component organization or biogenesis
developmental process
multicellular organismal process
signaling
reproductive process
multi-organism process
growth
reproduction
immune system process
biological adhesion
biological phase
rhythmic process
locomotion
cell killing
cell part
cell
organelle
membrane
organelle part
membrane part
macromolecular complex
extracellular region
cell junction
membrane-enclosed lumen
nucleoid
extracellular region part
virion
virion part
catalytic activity
binding
transporter activity
structural molecule activity
nucleic acid binding transcription factor activity
electron carrier activity
molecular transducer activity
enzyme regulator activity
antioxidant activity
receptor activity
protein binding transcription factor activity
guanyl-nucleotide exchange factor activity
nutrient reservoir activity
metallochaperone activity
translation regulator activity
protein tag
biological process
cellular component
molecular function


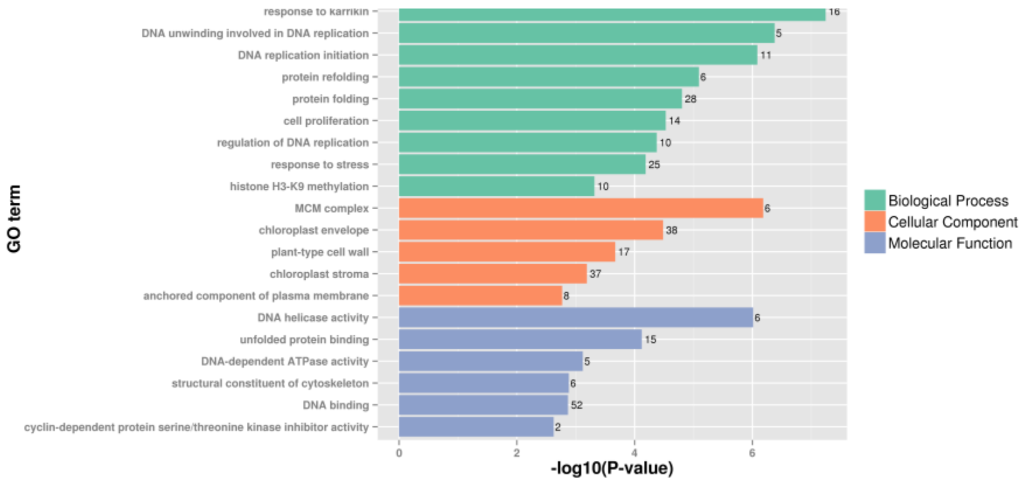

GO term
response to karrikin
DNA unwinding involved in DNA replication
DNA replication initiation
protein refolding
protein folding
cell proliferation
regulation of DNA replication
response to stress
histone H3-K9 methylation
MCM complex
chloroplast envelope
plant-type cell wall
chloroplast stroma
anchored component of plasma membrane
DNA helicase activity
unfolded protein binding
DNA-dependent ATPase activity
structural constituent of cytoskeleton
DNA binding
cyclin-dependent protein serine/threonine kinase inhibitor activity
16
5
11
6
28
14
10
25
10
6
38
17
37
8
6
15
5
6
52
2
0
2
4
6
-log10(P-value)
Biological Process
Cellular Component
Molecular Function

## Receptacle

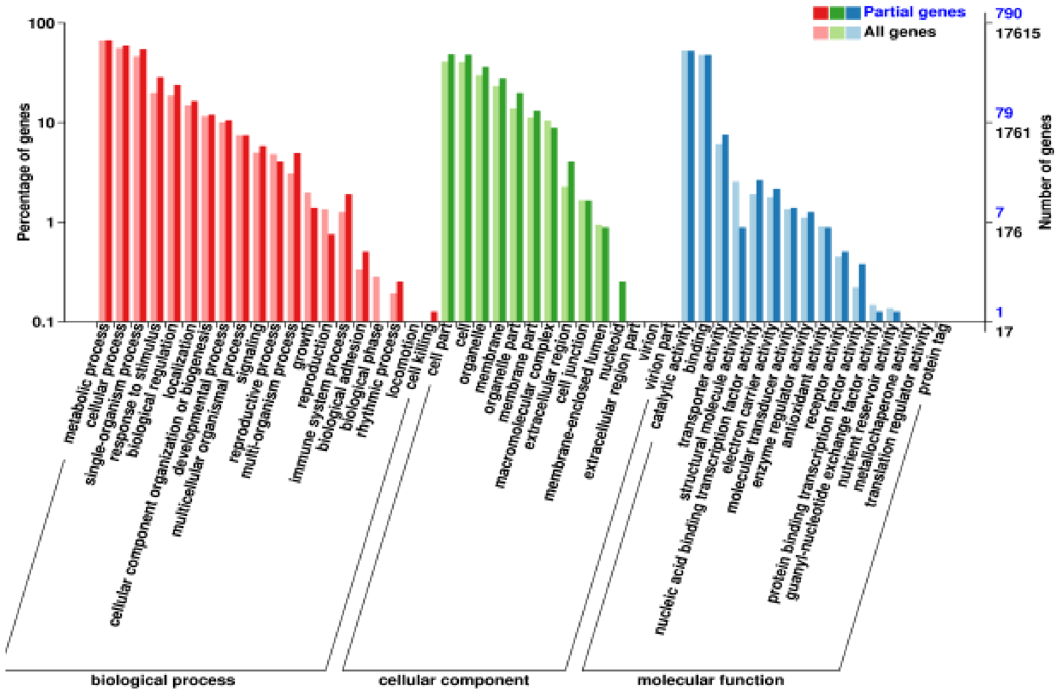
Partial genes
All genes
Percentage of genes
Number of genes
biological process
cellular component
molecular function

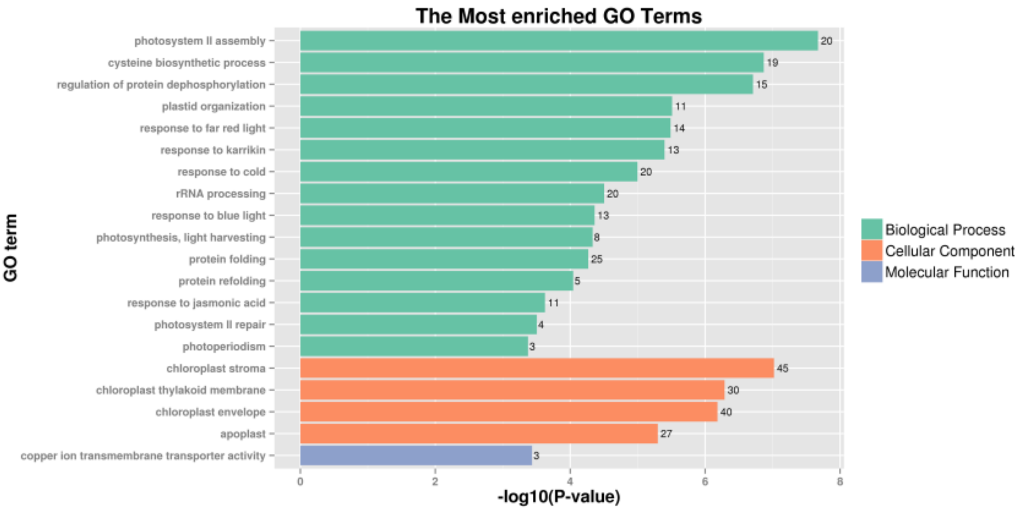
The Most enriched GO Terms
GO term
-log10(P-value)
Biological Process
Cellular Component
Molecular Function
photosystem II assembly
cysteine biosynthetic process
regulation of protein dephosphorylation
plastid organization
response to far red light
response to karrikin
response to cold
rRNA processing
response to blue light
photosynthesis, light harvesting
protein folding
protein refolding
response to jasmonic acid
photosystem II repair
photoperiodism
chloroplast stroma
chloroplast thylakoid membrane
chloroplast envelope
apoplast
copper ion transmembrane transporter activity

## Cotyledon

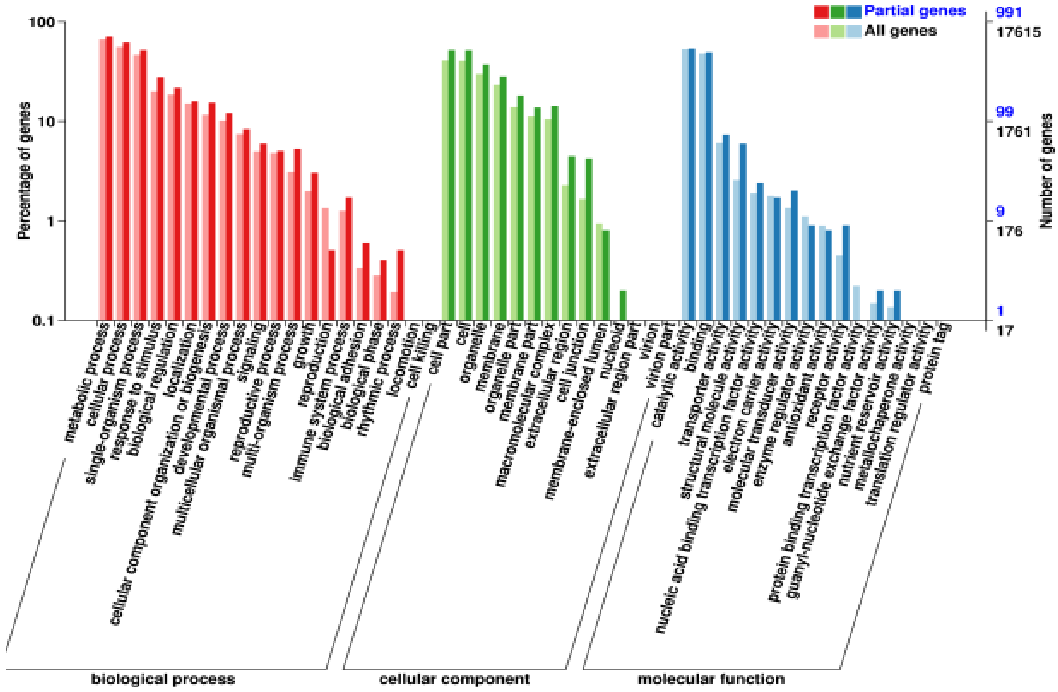
Partial genes
All genes
100
10
1
0.1
Percentage of genes
991
17615
99
1761
9
176
1
17
Number of genes
metabolic process
cellular process
single-organism process
response to stimulus
biological regulation
localization
cellular component organization or biogenesis
developmental process
multicellular organismal process
signaling
reproductive process
multi-organism process
growth
reproduction
immune system process
biological adhesion
biological phase
rhythmic process
locomotion
cell killing
cell part
cell
organelle
membrane
organelle part
membrane part
macromolecular complex
extracellular region
cell junction
membrane-enclosed lumen
nucleoid
extracellular region part
virion
virion part
catalytic activity
binding
transporter activity
structural molecule activity
nucleic acid binding transcription factor activity
electron carrier activity
molecular transducer activity
enzyme regulator activity
antioxidant activity
receptor activity
protein binding transcription factor activity
guanyl-nucleotide exchange factor activity
nutrient reservoir activity
metallochaperone activity
translation regulator activity
protein tag
biological process
cellular component
molecular function

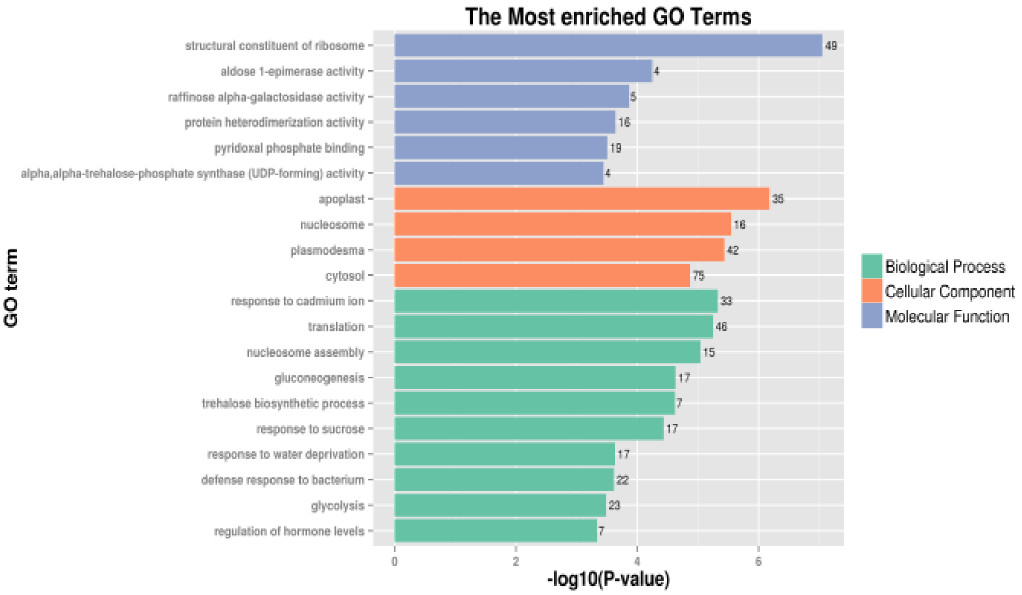
The Most enriched GO Terms
structural constituent of ribosome
aldose 1-epimerase activity
raffinose alpha-galactosidase activity
protein heterodimerization activity
pyridoxal phosphate binding
alpha,alpha-trehalose-phosphate synthase (UDP-forming) activity
apoplast
nucleosome
plasmodesma
cytosol
response to cadmium ion
translation
nucleosome assembly
gluconeogenesis
trehalose biosynthetic process
response to sucrose
response to water deprivation
defense response to bacterium
glycolysis
regulation of hormone levels
49
4
5
16
19
4
35
16
42
75
33
46
15
17
7
17
17
22
23
7
GO term
0
2
4
6
-log10(P-value)
Biological Process
Cellular Component
Molecular Function

# Elongation zone

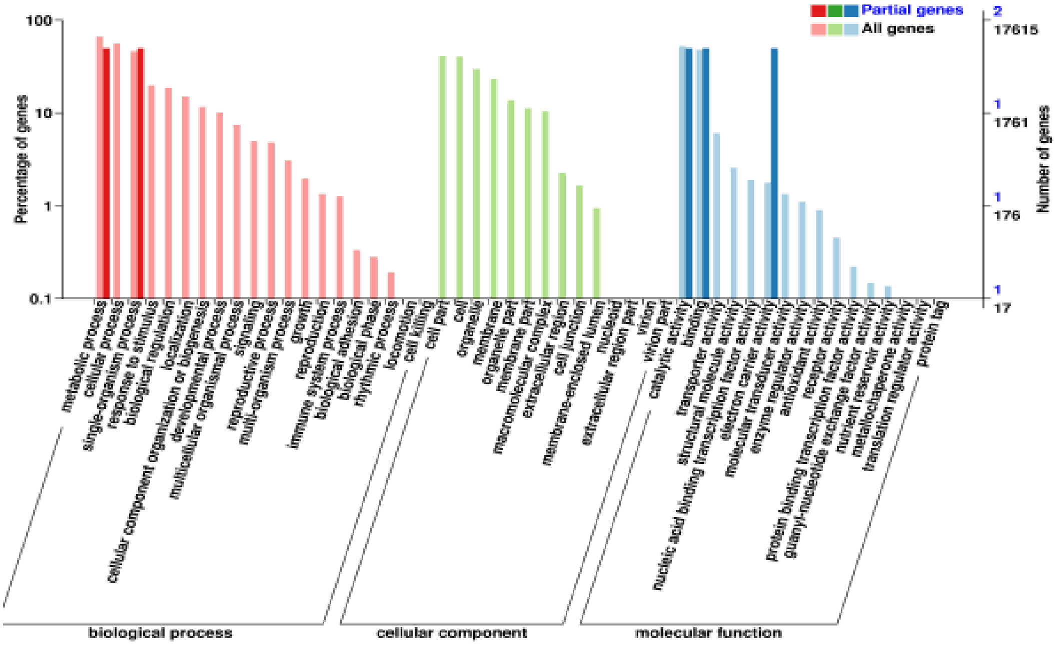


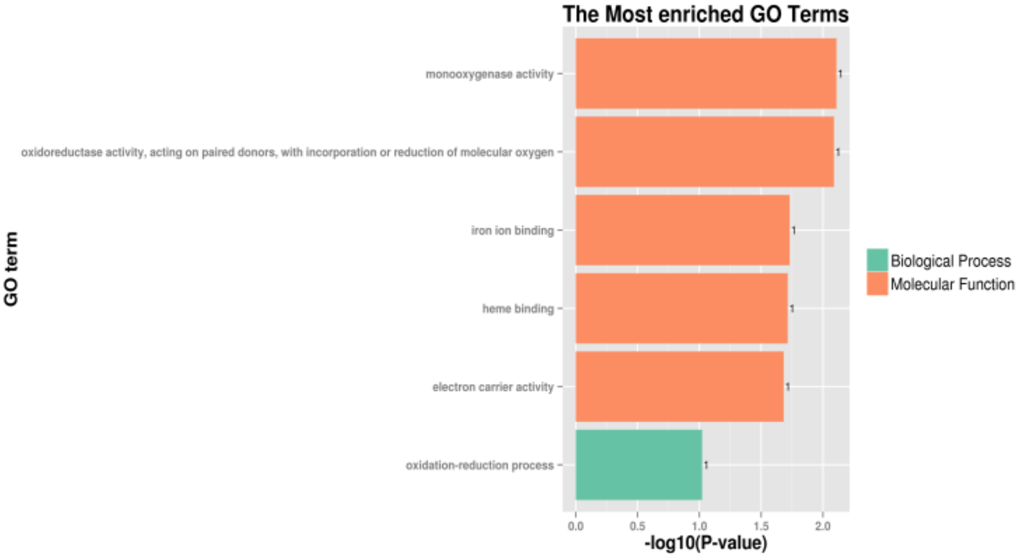

## Internode

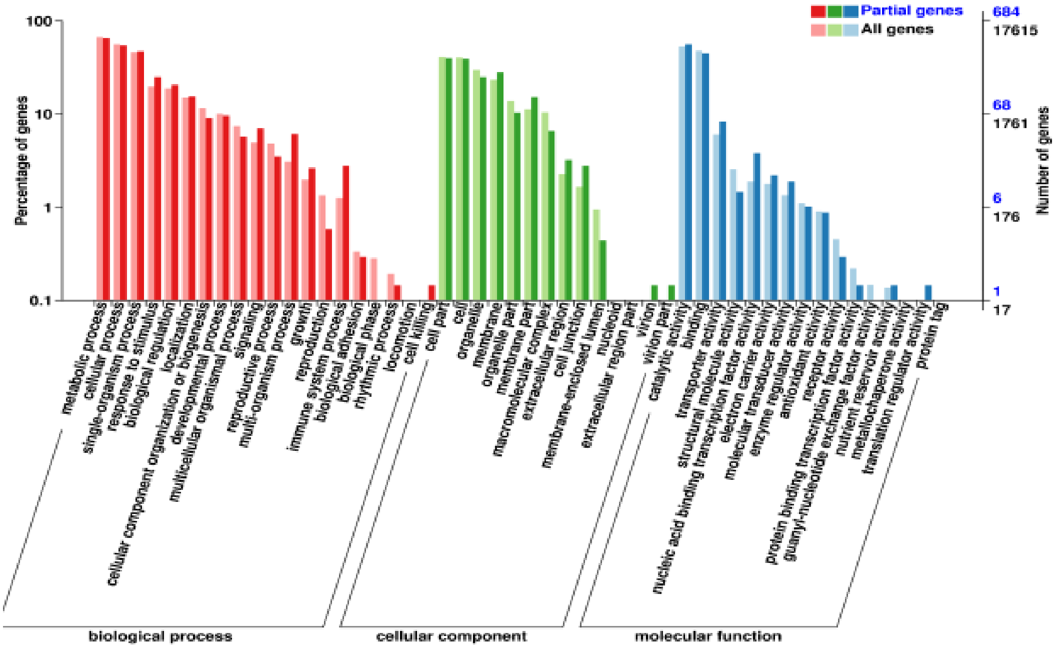

Partial genes
All genes
Percentage of genes
Number of genes
100
10
1
0.1
684
17615
68
1761
6
176
1
17
metabolic process
cellular process
single-organism process
response to stimulus
biological regulation
localization
cellular component organization or biogenesis
developmental process
multicellular organismal process
signaling
reproductive process
multi-organism process
growth
reproduction
immune system process
biological adhesion
biological phase
rhythmic process
locomotion
cell killing
cell part
cell
organelle
membrane
organelle part
membrane part
macromolecular complex
extracellular region
cell junction
membrane-enclosed lumen
nucleoid
extracellular region part
virion
virion part
catalytic activity
binding
transporter activity
structural molecule activity
nucleic acid binding transcription factor activity
electron carrier activity
molecular transducer activity
enzyme regulator activity
antioxidant activity
receptor activity
protein binding transcription factor activity
guanyl-nucleotide exchange factor activity
nutrient reservoir activity
metallochaperone activity
translation regulator activity
protein tag
biological process
cellular component
molecular function


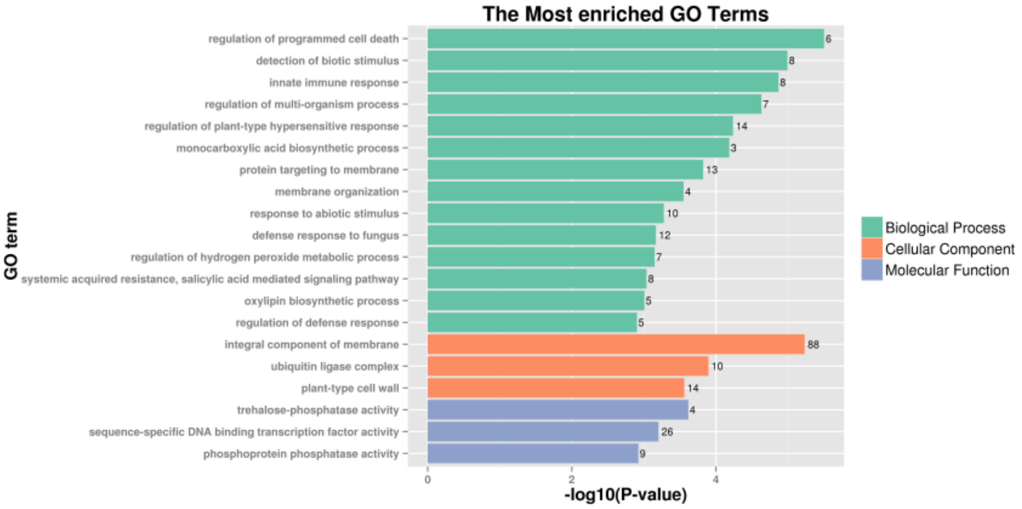

The Most enriched GO Terms
GO term
regulation of programmed cell death
detection of biotic stimulus
innate immune response
regulation of multi-organism process
regulation of plant-type hypersensitive response
monocarboxylic acid biosynthetic process
protein targeting to membrane
membrane organization
response to abiotic stimulus
defense response to fungus
regulation of hydrogen peroxide metabolic process
systemic acquired resistance, salicylic acid mediated signaling pathway
oxylipin biosynthetic process
regulation of defense response
integral component of membrane
ubiquitin ligase complex
plant-type cell wall
trehalose-phosphatase activity
sequence-specific DNA binding transcription factor activity
phosphoprotein phosphatase activity
6
8
8
7
14
3
13
4
10
12
7
8
5
5
88
10
14
4
26
9
0
2
4
-log10(P-value)
Biological Process
Cellular Component
Molecular Function

## Apical meristem

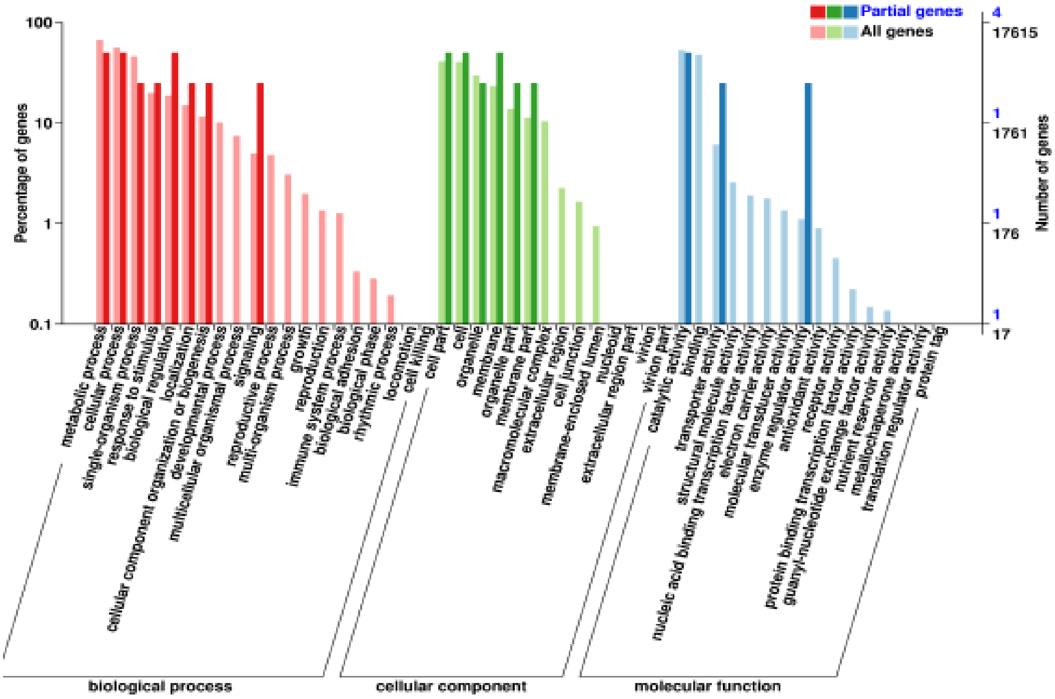


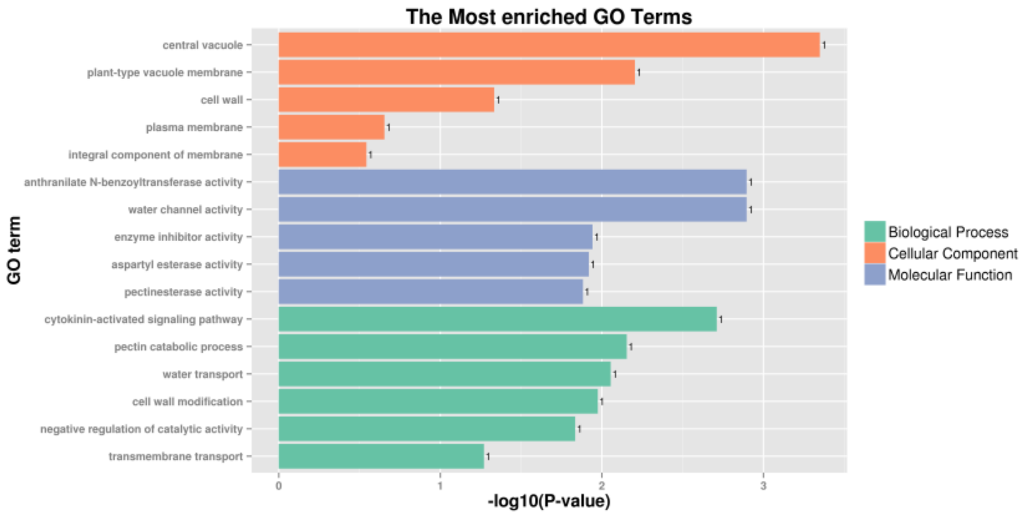

Root

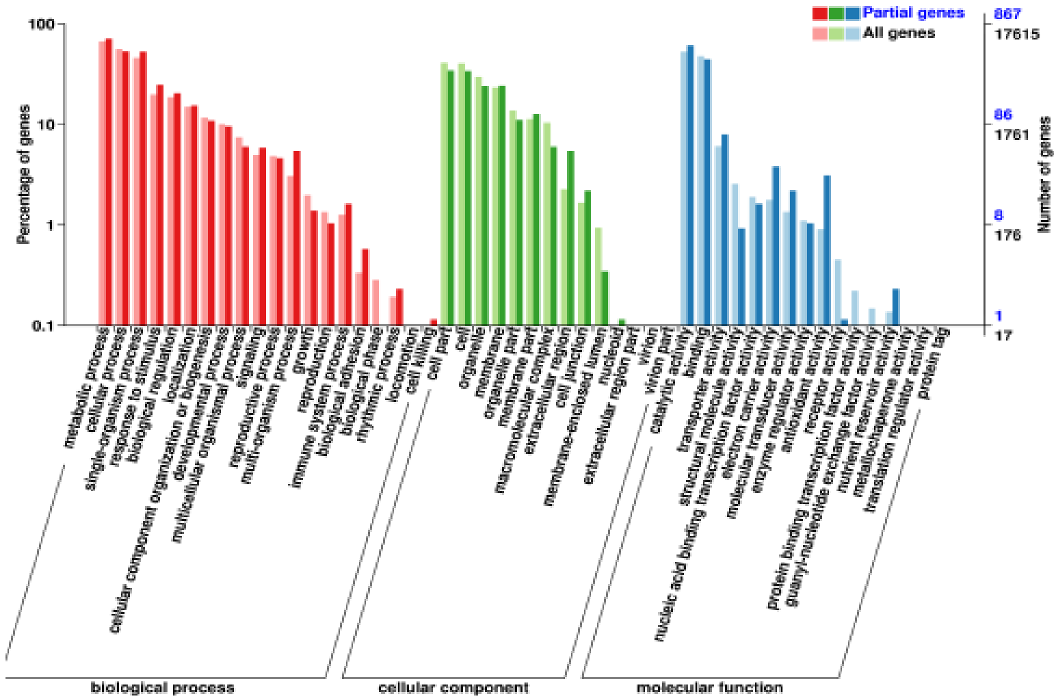
Partial genes
All genes
100
10
1
0.1
Percentage of genes
867
17615
86
1761
8
176
1
17
Number of genes
metabolic process
cellular process
single-organism process
response to stimulus
biological regulation
localization
cellular component organization or biogenesis
developmental process
multicellular organismal process
reproductive process
signaling
multi-organism process
growth
reproduction
immune system process
biological adhesion
biological phase
rhythmic process
locomotion
cell killing
cell part
cell
organelle
membrane
organelle part
membrane part
macromolecular complex
extracellular region
cell junction
membrane-enclosed lumen
nucleoid
extracellular region part
virion
virion part
catalytic activity
binding
transporter activity
structural molecule activity
nucleic acid binding transcription factor activity
electron carrier activity
molecular transducer activity
enzyme regulator activity
antioxidant activity
receptor activity
protein binding transcription factor activity
guanyl-nucleotide exchange factor activity
nutrient reservoir activity
metallochaperone activity
translation regulator activity
protein tag
biological process
cellular component
molecular function

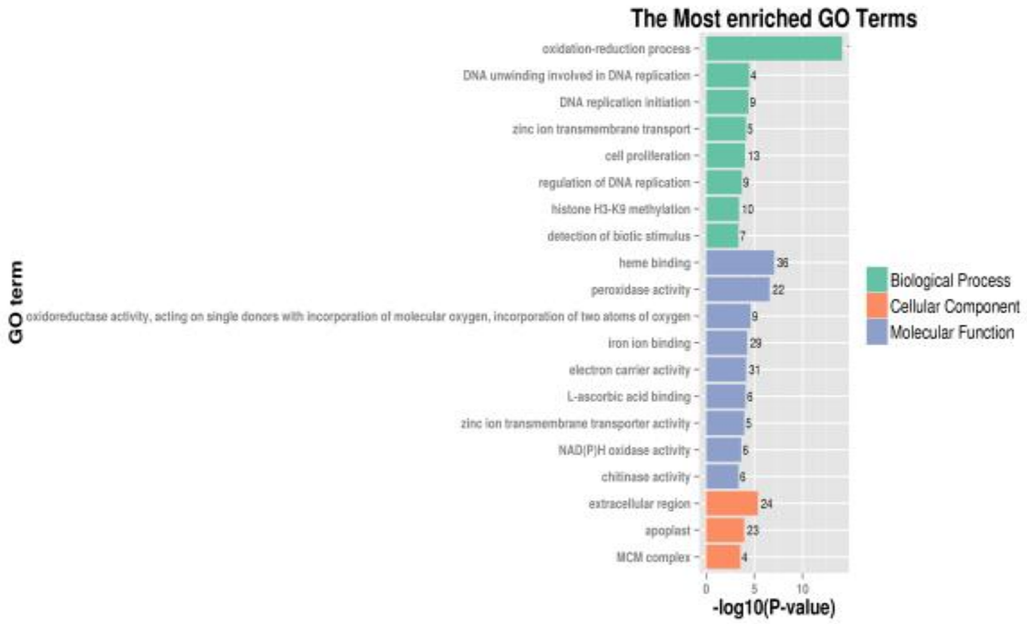
The Most enriched GO Terms
GO term
oxidation-reduction process
DNA unwinding involved in DNA replication
DNA replication initiation
zinc ion transmembrane transport
cell proliferation
regulation of DNA replication
histone H3-K9 methylation
detection of biotic stimulus
heme binding
peroxidase activity
oxidoreductase activity, acting on single donors with incorporation of molecular oxygen, incorporation of two atoms of oxygen
iron ion binding
electron carrier activity
L-ascorbic acid binding
zinc ion transmembrane transporter activity
NAD(P)H oxidase activity
chitinase activity
extracellular region
apoplast
MCM complex
4
9
5
13
9
10
7
36
22
9
29
31
6
5
6
6
24
23
4
0
5
10
-log10(P-value)
Biological Process
Cellular Component
Molecular Function

## Leaf

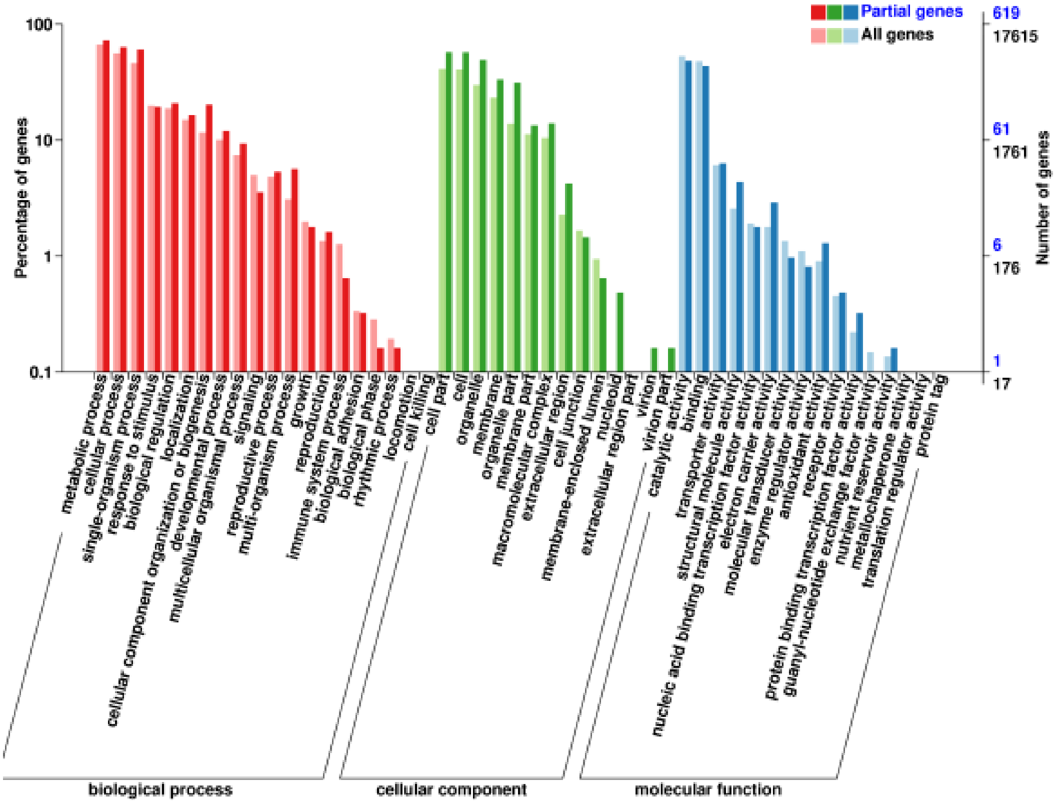

Partial genes
All genes
Percentage of genes
Number of genes
100
10
1
0.1
619
17615
61
1761
6
176
1
17
metabolic process
cellular process
single-organism process
response to stimulus
biological regulation
localization
cellular component organization or biogenesis
developmental process
multicellular organismal process
signaling
reproductive process
multi-organism process
growth
reproduction
immune system process
biological adhesion
biological phase
rhythmic process
locomotion
cell killing
cell part
cell
organelle
membrane
organelle part
membrane part
macromolecular complex
extracellular region
cell junction
membrane-enclosed lumen
nucleoid
extracellular region part
virion
virion part
catalytic activity
binding
transporter activity
structural molecule activity
nucleic acid binding transcription factor activity
electron carrier activity
molecular transducer activity
enzyme regulator activity
antioxidant activity
receptor activity
protein binding transcription factor activity
guanyl-nucleotide exchange factor activity
nutrient reservoir activity
metallochaperone activity
translation regulator activity
protein tag
biological process
cellular component
molecular function


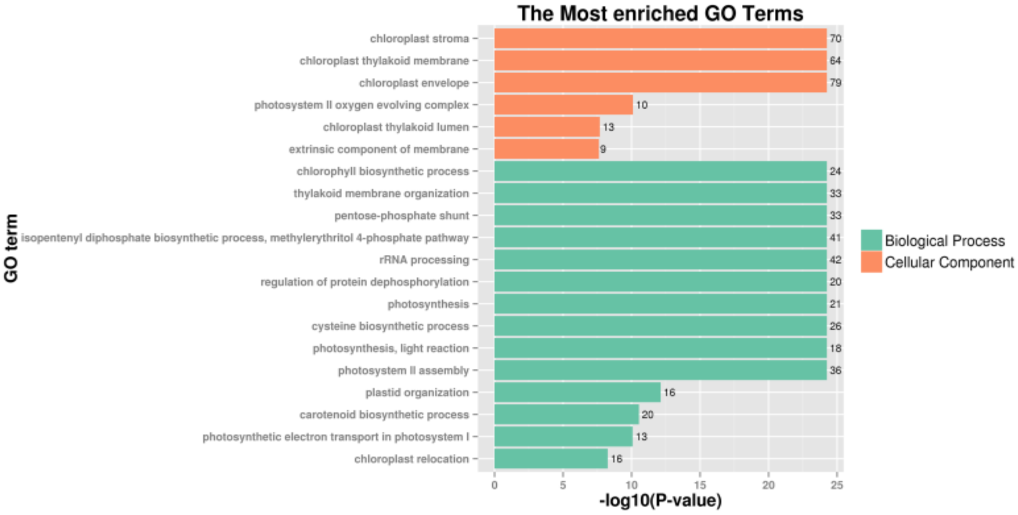

The Most enriched GO Terms
GO term
chloroplast stroma
chloroplast thylakoid membrane
chloroplast envelope
photosystem II oxygen evolving complex
chloroplast thylakoid lumen
extrinsic component of membrane
chlorophyll biosynthetic process
thylakoid membrane organization
pentose-phosphate shunt
isopentenyl diphosphate biosynthetic process, methylerythritol 4-phosphate pathway
rRNA processing
regulation of protein dephosphorylation
photosynthesis
cysteine biosynthetic process
photosynthesis, light reaction
photosystem II assembly
plastid organization
carotenoid biosynthetic process
photosynthetic electron transport in photosystem I
chloroplast relocation
70
64
79
10
13
9
24
33
33
41
42
20
21
26
18
36
16
20
13
16
0
5
10
15
20
25
-log10(P-value)
Biological Process
Cellular Component

## Petal

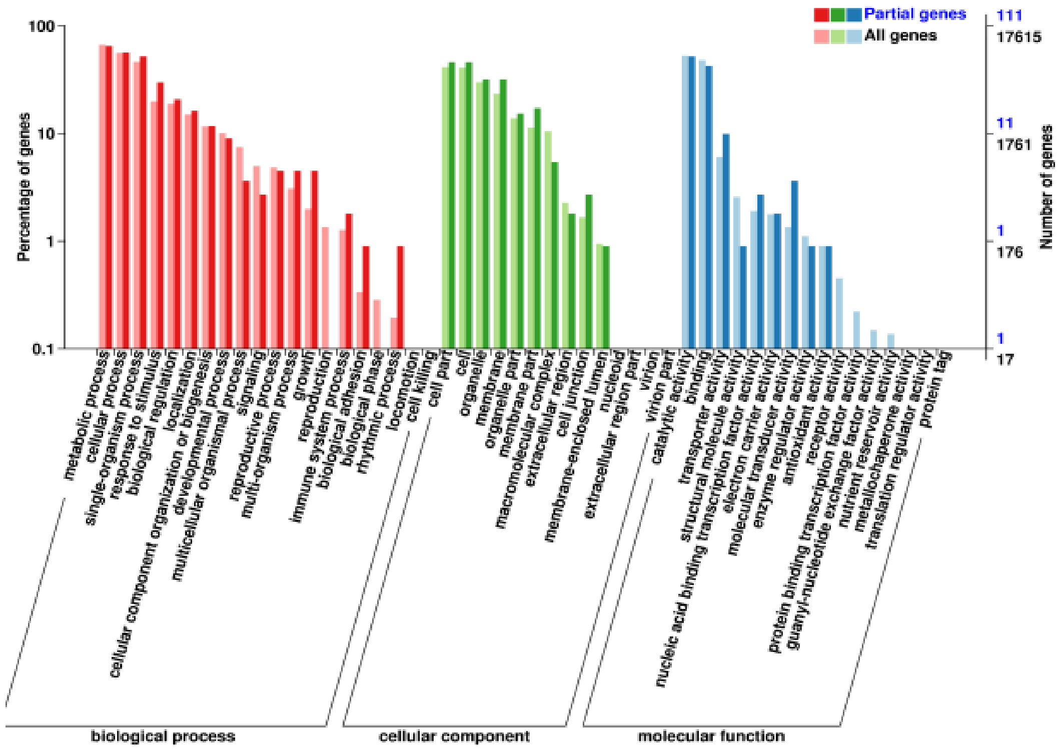

Partial genes
All genes
Percentage of genes
Number of genes
100
10
1
0.1
111
17615
11
1761
1
176
1
17
metabolic process
cellular process
single-organism process
response to stimulus
biological regulation
localization
cellular component organization or biogenesis
developmental process
multicellular organismal process
signaling
reproductive process
multi-organism process
growth
reproduction
immune system process
biological adhesion
biological phase
rhythmic process
locomotion
cell killing
cell part
cell
organelle
membrane
organelle part
membrane part
macromolecular complex
extracellular region
cell junction
membrane-enclosed lumen
nucleoid
extracellular region part
virion
virion part
catalytic activity
binding
transporter activity
structural molecule activity
nucleic acid binding transcription factor activity
electron carrier activity
molecular transducer activity
enzyme regulator activity
antioxidant activity
receptor activity
protein binding transcription factor activity
guanyl-nucleotide exchange factor activity
nutrient reservoir activity
metallochaperone activity
translation regulator activity
protein tag
biological process
cellular component
molecular function


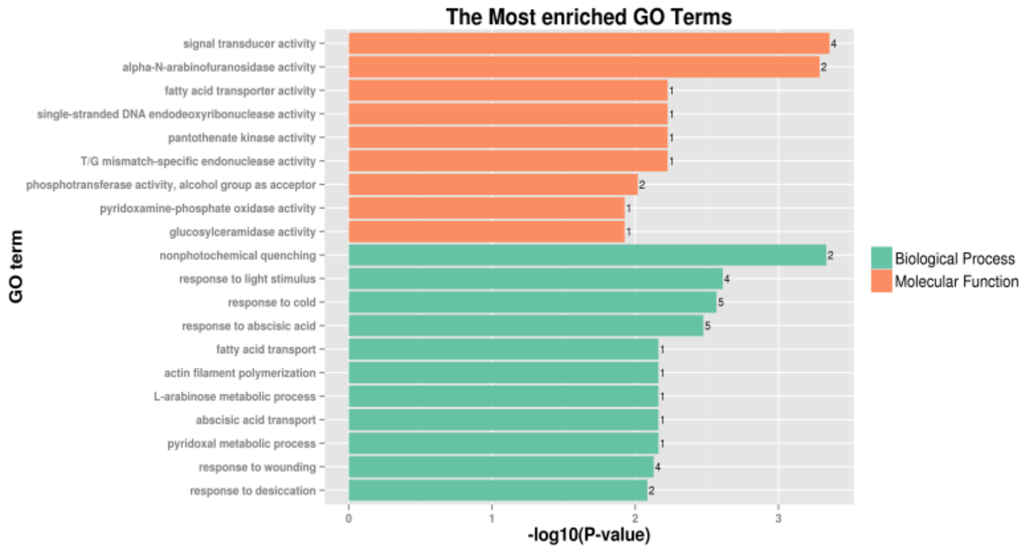

The Most enriched GO Terms
GO term
signal transducer activity
alpha-N-arabinofuranosidase activity
fatty acid transporter activity
single-stranded DNA endodeoxyribonuclease activity
pantothenate kinase activity
T/G mismatch-specific endonuclease activity
phosphotransferase activity, alcohol group as acceptor
pyridoxamine-phosphate oxidase activity
glucosylceramidase activity
nonphotochemical quenching
response to light stimulus
response to cold
response to abscisic acid
fatty acid transport
actin filament polymerization
L-arabinose metabolic process
abscisic acid transport
pyridoxal metabolic process
response to wounding
response to desiccation
4
2
1
1
1
1
2
1
1
2
4
5
5
1
1
1
1
1
4
2
0
1
2
3
-log10(P-value)
Biological Process
Molecular Function

## Petiole

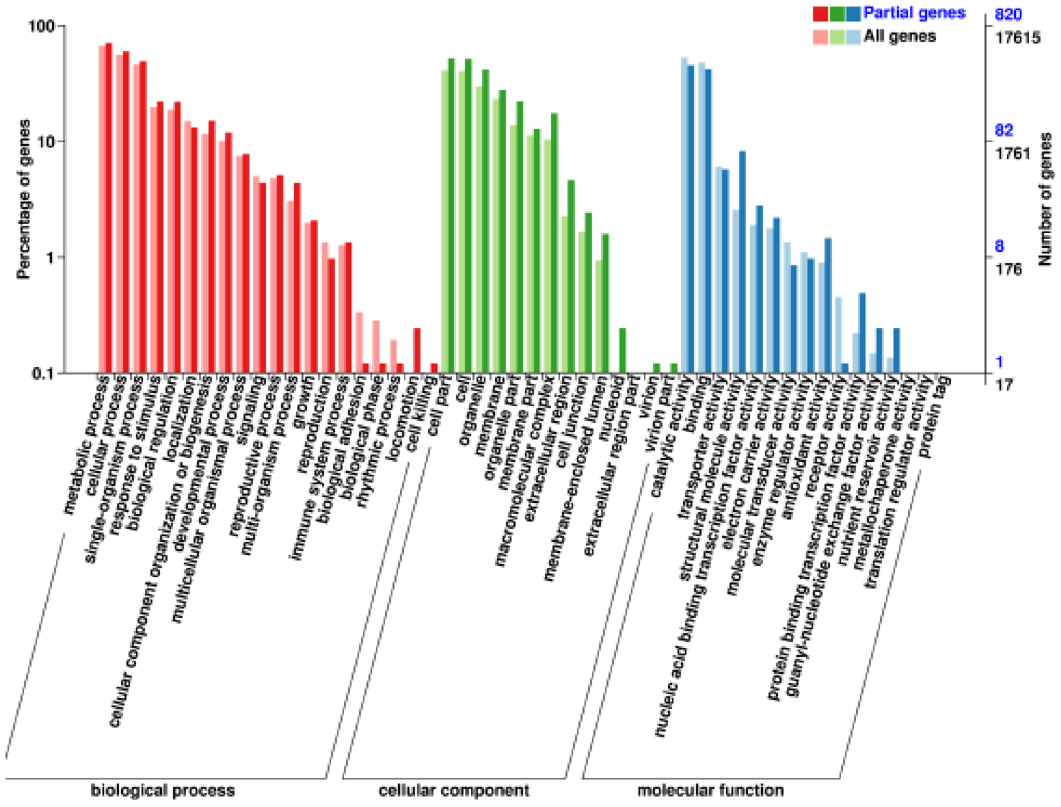

Partial genes
All genes
Percentage of genes
Number of genes
100
10
1
0.1
820
17615
82
1761
8
176
1
17
metabolic process
cellular process
single-organism process
response to stimulus
biological regulation
localization
cellular component organization or biogenesis
developmental process
multicellular organismal process
signaling
reproductive process
multi-organism process
growth
reproduction
immune system process
biological adhesion
biological phase
rhythmic process
locomotion
cell killing
cell part
cell
organelle
membrane
organelle part
membrane part
macromolecular complex
extracellular region
cell junction
membrane-enclosed lumen
nucleoid
extracellular region part
virion
virion part
catalytic activity
binding
transporter activity
structural molecule activity
nucleic acid binding transcription factor activity
electron carrier activity
molecular transducer activity
enzyme regulator activity
antioxidant activity
receptor activity
protein binding transcription factor activity
guanyl-nucleotide exchange factor activity
nutrient reservoir activity
metallochaperone activity
translation regulator activity
protein tag
biological process
cellular component
molecular function


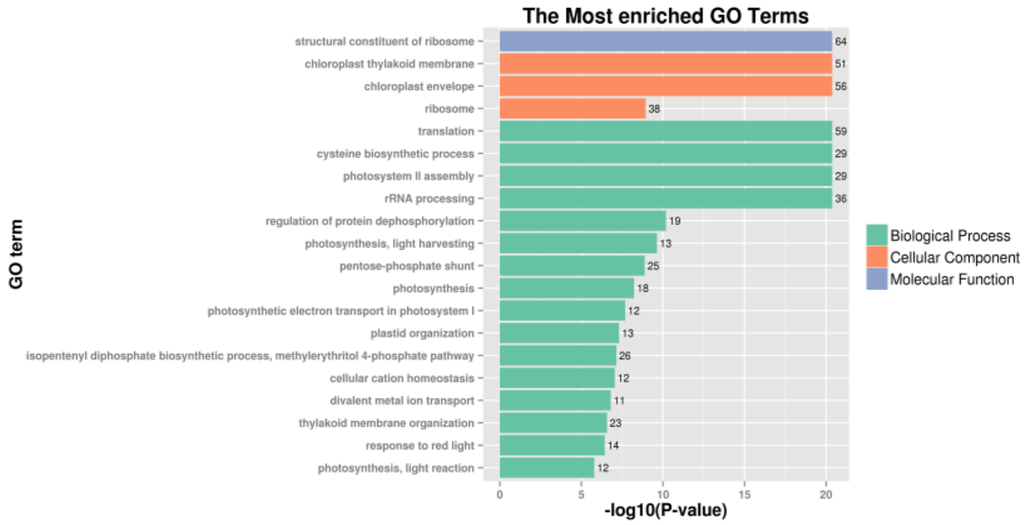

The Most enriched GO Terms
GO term
structural constituent of ribosome
chloroplast thylakoid membrane
chloroplast envelope
ribosome
translation
cysteine biosynthetic process
photosystem II assembly
rRNA processing
regulation of protein dephosphorylation
photosynthesis, light harvesting
pentose-phosphate shunt
photosynthesis
photosynthetic electron transport in photosystem I
plastid organization
isopentenyl diphosphate biosynthetic process, methylerythritol 4-phosphate pathway
cellular cation homeostasis
divalent metal ion transport
thylakoid membrane organization
response to red light
photosynthesis, light reaction
64
51
56
38
59
29
29
36
19
13
25
18
12
13
26
12
11
23
14
12
0
5
10
15
20
-log10(P-value)
Biological Process
Cellular Component
Molecular Function

## Seed coat

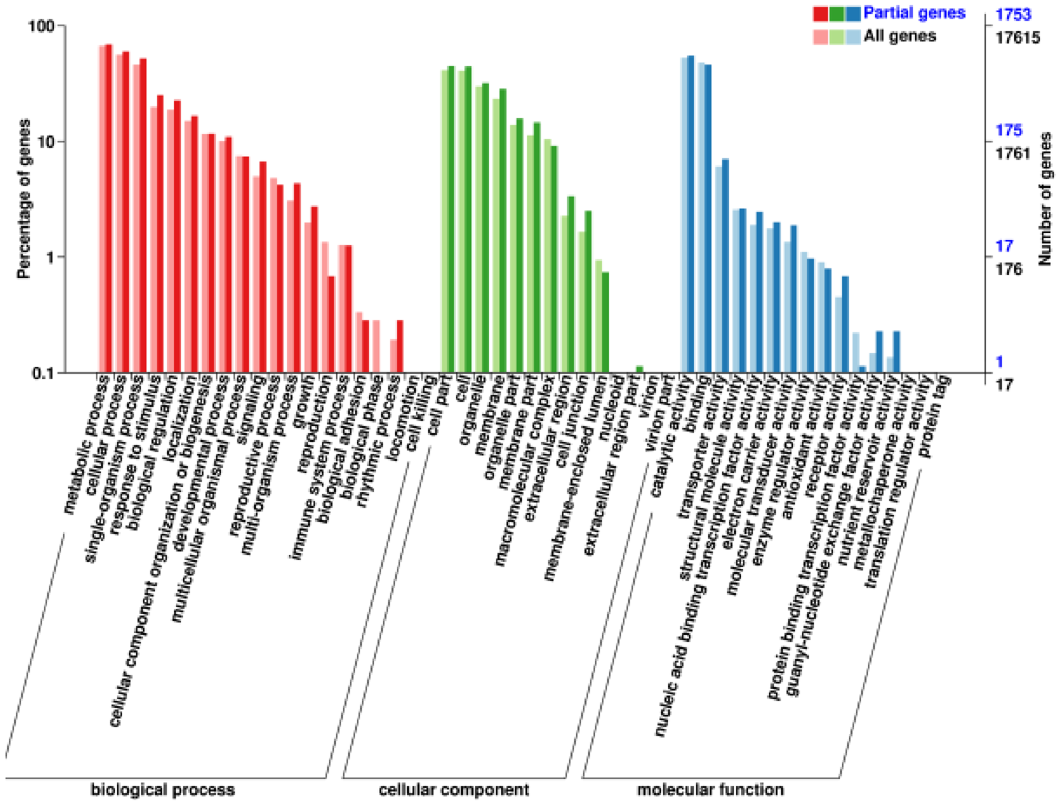

Partial genes
All genes
1753
17615
175
1761
17
176
1
17
100
10
1
0.1
Percentage of genes
Number of genes
metabolic process
cellular process
single-organism process
response to stimulus
biological regulation
localization
cellular component organization or biogenesis
developmental process
multicellular organismal process
signaling
reproductive process
multi-organism process
growth
reproduction
immune system process
biological adhesion
biological phase
rhythmic process
locomotion
cell killing
cell part
cell
organelle
membrane
organelle part
membrane part
macromolecular complex
extracellular region
cell junction
membrane-enclosed lumen
nucleoid
extracellular region part
virion
virion part
catalytic activity
binding
transporter activity
structural molecule activity
nucleic acid binding transcription factor activity
electron carrier activity
molecular transducer activity
enzyme regulator activity
antioxidant activity
receptor activity
protein binding transcription factor activity
guanyl-nucleotide exchange factor activity
nutrient reservoir activity
metallochaperone activity
translation regulator activity
protein tag
biological process
cellular component
molecular function


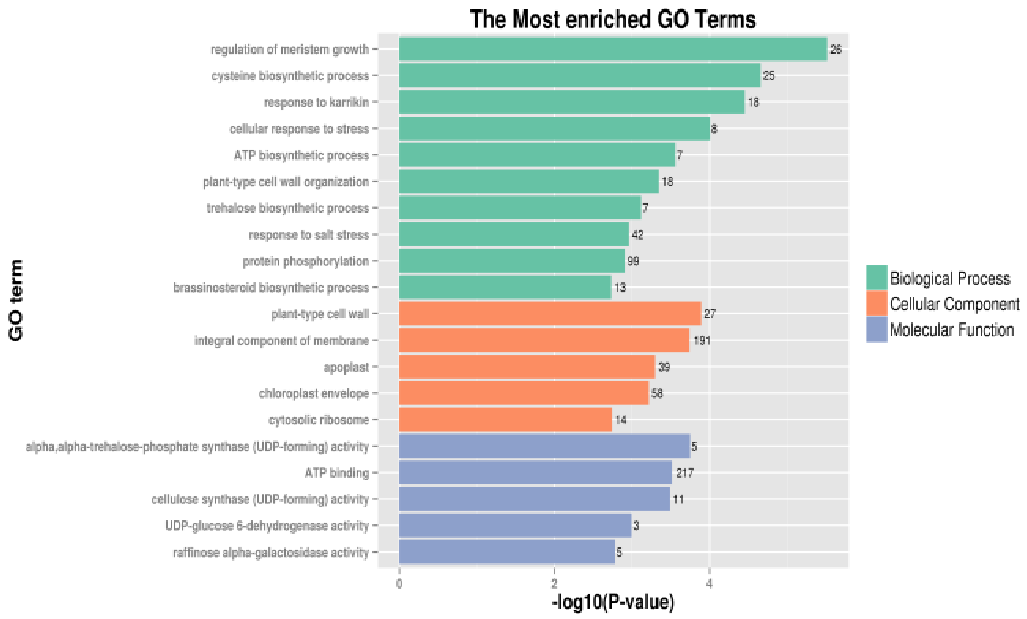

The Most enriched GO Terms
GO term
regulation of meristem growth
cysteine biosynthetic process
response to karrikin
cellular response to stress
ATP biosynthetic process
plant-type cell wall organization
trehalose biosynthetic process
response to salt stress
protein phosphorylation
brassinosteroid biosynthetic process
plant-type cell wall
integral component of membrane
apoplast
chloroplast envelope
cytosolic ribosome
alpha,alpha-trehalose-phosphate synthase (UDP-forming) activity
ATP binding
cellulose synthase (UDP-forming) activity
UDP-glucose 6-dehydrogenase activity
raffinose alpha-galactosidase activity
26
25
18
8
7
18
7
42
99
13
27
191
39
58
14
5
217
11
3
5
0
2
4
-log10(P-value)
Biological Process
Cellular Component
Molecular Function

**Fig. S10** Heatmap of filtered isoforms in 12 tissues. The expression level is displayed with different color based on log2 (FPKM)

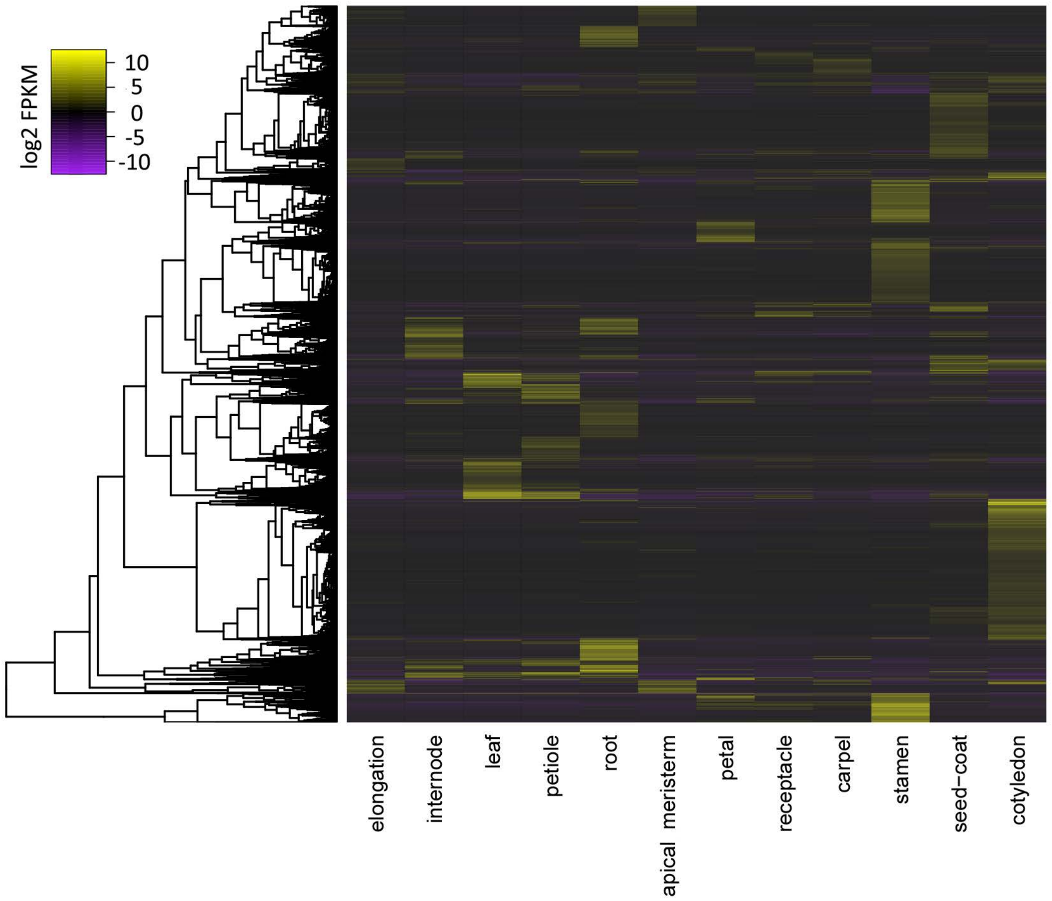

**Fig.S11 Clusters of filtered isoforms. Twelve clusters were classified based on isoforms expression pattern.**

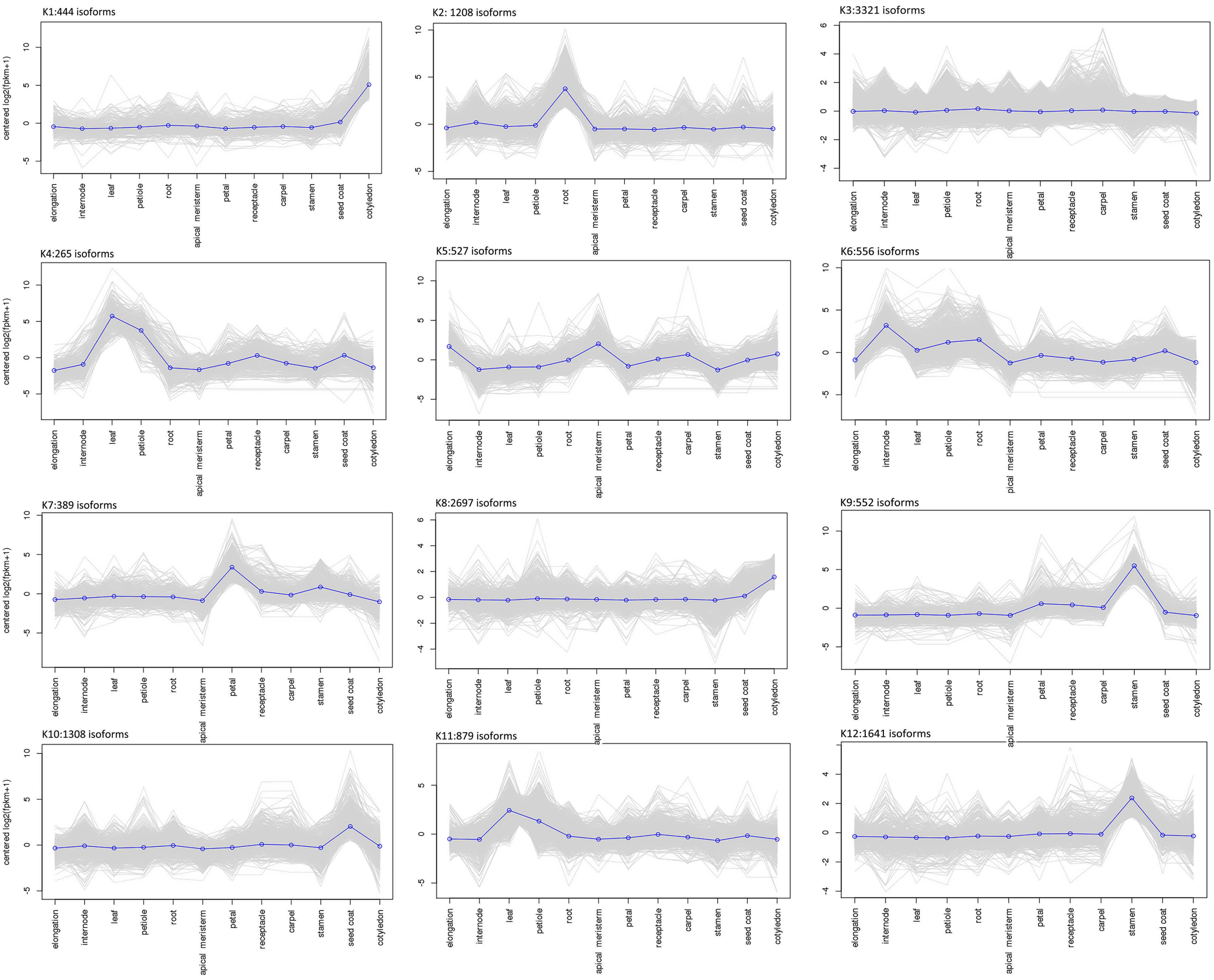

**Fig. S12** Distribution of differential expressed genes and isoforms between different floral tissues.

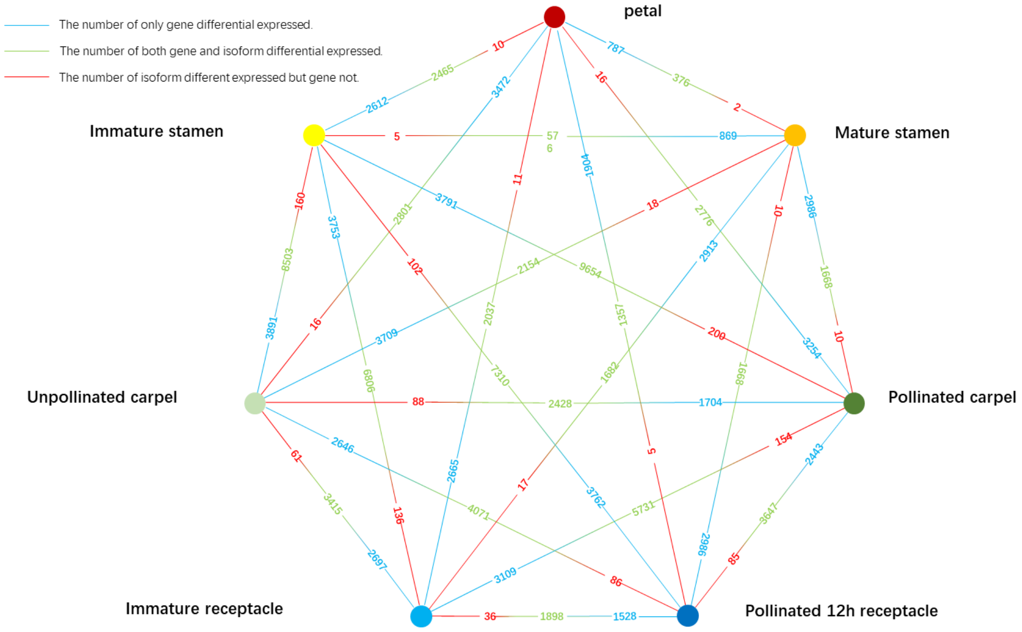

**Fig. S13** Phylogenetic analysis of MADS-box genes from lotus and Arabidopsis. The green dots before the gene ID represent lotus genes, and the red ones represent Arabidopsis genes. Genes inside the red, blue, green and yellow rectangles are A, B, C and E genes in the “ABCE” model, respectively.

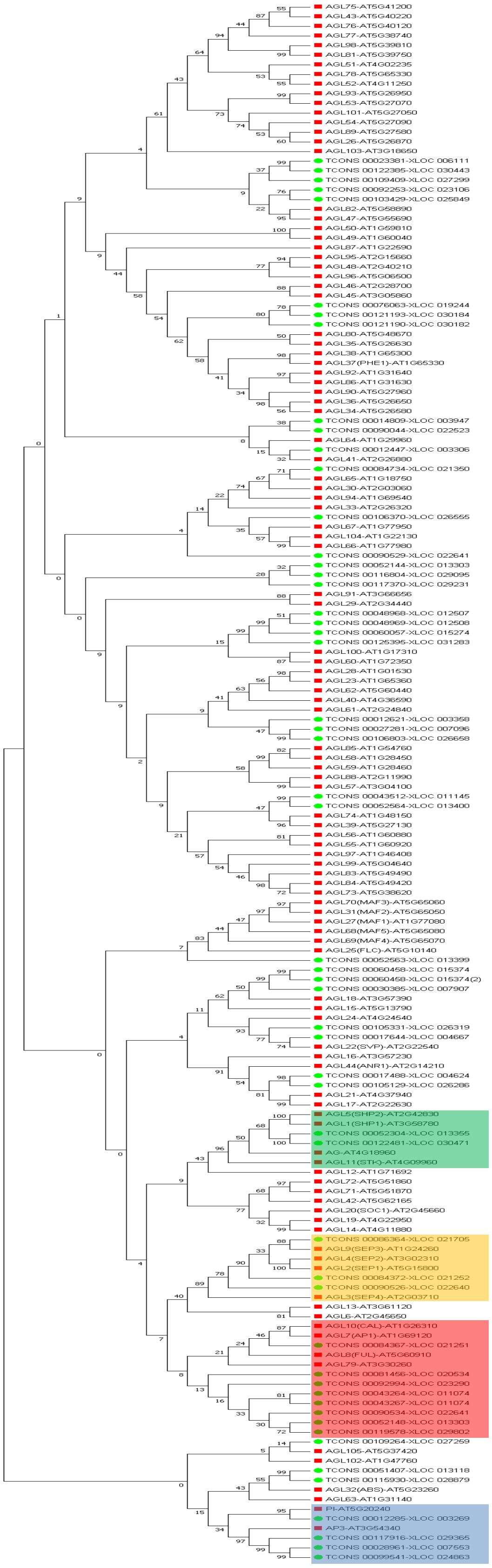

**Fig. S14** Heatmap showing the expression of MADS-box gene in different floral tissues of lotus. Genes inside the red, blue, green and yellow rectangles are A, B, C and E genes in the "ABCE" model, respectively.

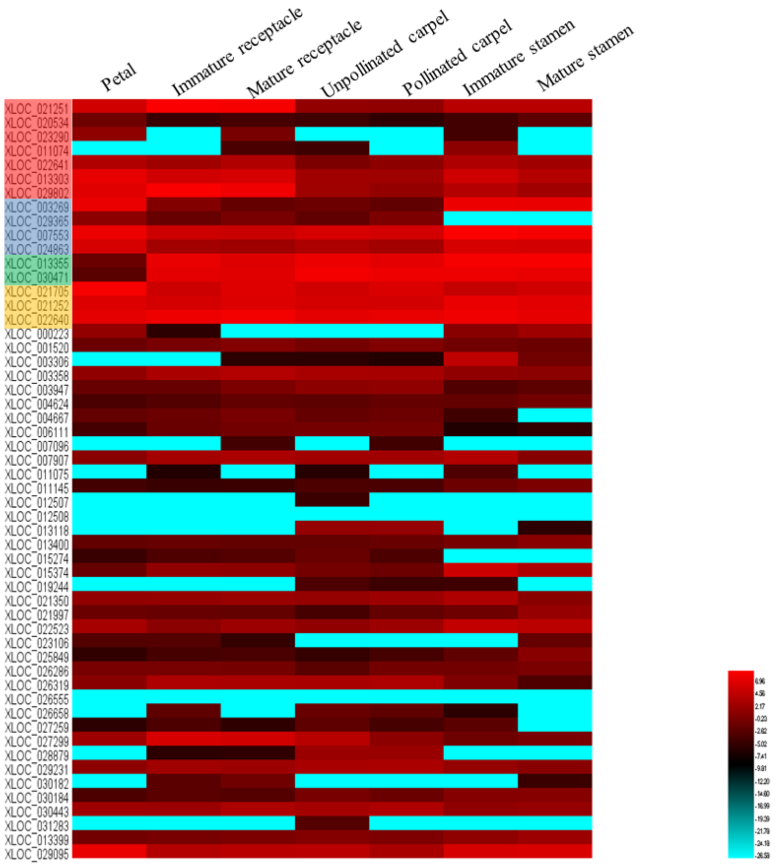

Supplement: dsz010_Supplementary_Data [file dsz010_supplementary_data.zip › dsz010-Suppl_data/Supplementary Figures.pdf]
